# Addressing the “minimum parking” problem for on-demand mobility

**DOI:** 10.1038/s41598-020-71867-1

**Published:** 2020-10-08

**Authors:** Dániel Kondor, Paolo Santi, Diem-Trinh Le, Xiaohu Zhang, Adam Millard-Ball, Carlo Ratti

**Affiliations:** 1 Singapore-MIT Alliance for Research and Technology, Singapore, 138602 Singapore; 2 Senseable City Laboratory, MIT, Cambridge, MA 02139 USA; 3 Istituto di Informatica e Telematica del CNR, Pisa, Italy; 4 University of California Santa Cruz, Santa Cruz, CA USA

**Keywords:** Socioeconomic scenarios, Scientific data

## Abstract

Parking infrastructure is pervasive and occupies large swaths of land in cities. However, on-demand (OD) mobility has started reducing parking needs in urban areas around the world. This trend is expected to grow significantly with the advent of autonomous driving, which might render on-demand mobility predominant. Recent studies have started looking at expected parking reductions with on-demand mobility, but a systematic framework is still lacking. In this paper, we apply a data-driven methodology based on shareability networks to address what we call the “minimum parking” problem: what is the minimum parking infrastructure needed in a city for given on-demand mobility needs? While solving the problem, we also identify a critical tradeoff between two public policy goals: less parking means increased vehicle travel from deadheading between trips. By applying our methodology to the city of Singapore we discover that parking infrastructure reduction of up to 86% is possible, but at the expense of a 24% increase in traffic measured as vehicle kilometers travelled (VKT). However, a more modest 57% reduction in parking is achievable with only a 1.3% increase in VKT. We find that the tradeoff between parking and traffic obeys an inverse exponential law which is invariant with the size of the vehicle fleet. Finally, we analyze parking requirements due to passenger pick-ups and show that increasing convenience produces a substantial increase in parking for passenger pickup/dropoff. The above findings can inform policy-makers, mobility operators, and society at large on the tradeoffs required in the transition towards pervasive on-demand mobility.

## Introduction

Cities currently devote a large amount of space and resources to provide parking, primarily used by private cars that are idle 95% of the time^1^. For example, in Los Angeles County, where there are 3.3 parking spaces per car, the total area of parking spaces is equal to 14% of total incorporated land area and is 1.4 times larger than the total area used by roads^2^. In dense city centers, parking can account for an even larger share—the total floor area dedicated to parking is between 25 and 81% of land area^3^ and can be larger than the floor area of office or retail use that it serves^4^. Using floor area as a metric counts each level of a parking garage separately, and so the spatial footprint of parking is less, but it can still account for over 5% of total urban land areas^5,6^. Similar levels of parking are mandated in many Asian cities^7^. Provision of parking contributes to urban sprawl and high energy use associated with private car use and building and maintaining an excessive road infrastructure^3,8,9^.

However, changes are starting to be apparent with the increased popularity of shared mobility services provided by on-demand vehicle (OV) fleets, such as ridehailing services, which can increase vehicle on-road time and reduce parking needs^10–12^. Further opportunities are foreseen with the gradual transition to autonomous vehicles (AVs)^13–15^, which are expected to reduce the number of privately-owned cars and further popularize shared mobility^16–18^ due to being more cost-effective than both taxis and private vehicles^1,19,20^. OV fleets could reduce a city’s parking needs through several mechanisms. First, thanks to vehicle and/or ride sharing, OVs are expected to reduce the size of the vehicle fleet by 40–90%^1,21–24^, accompanied by similar reduction in the demand for parking^25–28^. Furthermore, OVs have no need to park at their destination, and can return home, park remotely, or even cruise (circle) around^29,30^, resulting in increased utilization of a smaller amount of parking. AVs can further reduce the spatial footprint of parking facilities by exploiting better maneuvering capabilities and the fact that individual vehicles need not be accessible to humans when parked^31–33^. While autonomous mobility is still forthcoming, most of the benefits related to the use of shared, on-demand mobility could be realized today. For this reason, in this study, we use the generic term OV, with the understanding that the model and results presented here apply to both autonomous and chauffeured vehicles, but might only be realized with AVs due to economic reasons. Nevertheless, we note that cities have policy tools to enforce more optimal fleet operations for OV vehicles today^50,51^. We do not account for the potentional additional benefits due to better maneuverability of AVs mentioned above, that could further decrease the physical footprint of parking spaces.

Despite the initial studies mentioned above, a precise mathematical solution to the minimum parking problem is still missing. We can phrase the problem as follows: given a number *NT* of private vehicle trips, what is the minimum parking infrastructure needed to support this demand? In this paper, we use shareability networks and related optimal dispatching algorithms^24,28^ and derive the minimum number of parking spots $NP_{ov}$ needed in a city, for a given size $NV_{ov}$ of the fleet used to serve the *NT* trips. Clearly, on demand vehicles could be moving all the time, reducing the number of parking spots to zero. Hence, we introduce a parameter $r_{{\max }}$ that bounds the number of empty kilometers a vehicle can drive in-between trips, and show how $NP_{ov}$ varies as a function of $r_{{\max }}$. Finally, we measure the total additional travel distance $TD_{extra}$ needed to serve the *NT* trips with $NV_{ov}$ vehicles and $NP_{ov}$ parking spots, and formally characterize a tradeoff between the three quantities at stake (number of vehicles, parking infrastructure, and traveled distance) in a case study of the city of Singapore.

## Results

### Parking demand for OD mobility

**Figure 1:**
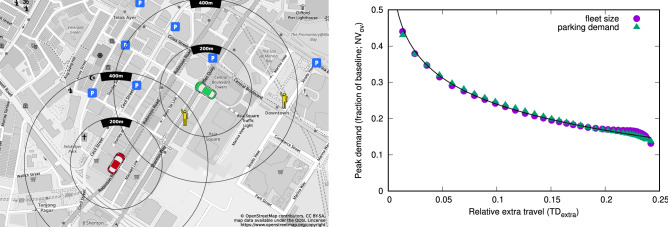
Tradeoff between parking demand and vehicle travel. The left panel shows an illustration of the problem and the process used to estimate parking requirements. It shows a simplified case with two on-demand vehicles, several available parking spaces and two passengers requesting a ride: the red vehicle is just dropping off a passenger and would need to find parking or a next passenger, while the green vehicle is already parked and would be available for new trips. We show search radiuses of $r_{{\max }} = 200\;{\text{m}}$ and $400\,\mathrm {m}$ for each vehicle; note that these values are used here for illustration purposes only as these are smaller than the values of $r_{{\max }}$ used in the actual simulations. Also, this illustration shows Euclidean distances, while the actual simulations use real distances calculated along the road network. For $r_{{\max }} = 200\,\mathrm {m}$, the red vehicle cannot find any parking, while the green vehicle can only serve one of the passengers. In this case, we would need to add further parking for the red vehicle and one additional vehicle to serve the passenger farther away. For $r_{{\max }} = 400\,\mathrm {m}$, the red vehicle has a choice of parking or serving one passenger, while the green vehicle could serve either passenger. Performing a maximum matching in this case will assign the two vehicles to the two passengers, thus resulting in a solution with less vehicles and parking, but more extra travel. On the right panel, we show estimation results with different values of $r_{{\max }}$, ranging from $500\,\mathrm {m}$ to unlimited, with each point corresponding to one possible value. We display relative demand for parking and vehicle fleet size as compared to our current estimate of available parking and fleet size on the *y*-axis as a function of additional VKT on the *x*-axis. The fitted line suggests that increase in VKT grows exponentially as a function of decrease in fleet size and parking requirements (see SI for discussion). Left panel was edited with Inkscape, version 0.92.3, available at https://inkscape.org/; right panel created with Gnuplot, version 5.2, available at http://gnuplot.info/.

For the present study, we need to capture overall vehicular mobility demand in Singapore. We use data from SimMobility, probably the most precise and comprehensive simulator for urban mobility which incorporates a detailed model of people’s movements in Singapore^34^. We concentrate on the trips made in private vehicles and investigate the scenario in which all of these trips are served by on-demand vehicles instead. Investigating changes in mode choice due to availability of OV or AV as a travel option is an important question, but is beyond the scope of the current work as it has been studied extensively elsewhere^16,18,35–38^. Our methodology could however be easily scaled and applied to cases with other assumptions of travel demand^28^. We use a methodology based on bipartite matching of vehicles to trips and parking spaces to arrive at an estimate of $NV_{ov}$, $NP_{ov}$, and $TD_{extra}$^24,28^—see Fig. 1 (left panel) for an illustration and the “Methods” and “Supplementary Material” for a detailed description. We compare our results to an estimation of parking supply based on land-use data, parking requirements and constraints based on the SimMobility trip dataset, that yields a total of 1.37 million parking spaces, of which at least 25,740 are curb parking (see the Supplementary Material Sect. 4 and Table S4 for the description of parking types and estimation methodology).

The right panel in Fig. 1 reports the main results of the analysis. We find that parking could be reduced by as much as 86% from the current estimate of around 1.37 million to about 189 thousand. At the same time, the number of vehicles would be reduced by a similar ratio, from around 676 thousand to below 89 thousand.

However, savings in parking come at a cost: in our minimum parking scenario, the OV fleet has about 24.2% more travel (total VKT) than the baseline case of people making the same trips in private vehicles. (Note that this estimate relies on the assumption that private car users can find parking exactly at their destination which is not true in all cases. Thus, we see our estimate of VKT increase as an upper bound.) We note that in this paper, we use VKT as a proxy for negative externalities of vehicular travel and thus view the increase in VKT as a measure of potential negative outcomes from an adoption of OVs; we acknowledge that the relationship between VKT and pollution, energy use, traffic congestion, accidents and noise can be non-linear and will be affected by independent factors such as vehicle propulsion and form factor, driving style and safety measures for both chauffeur-driven and autonomous vehicles^39,40^. Nevertheless, our results regarding the increase in total VKT highlight a tradeoff between parking reduction and increased vehicle travel. We control this tradeoff via the parameter $r_{{\max }}$, that determines the maximum distance OVs are allowed to travel to reach parking or the next passenger. While in practice, $r_{{\max }}$ will be a parameter determined by economics of fleet operations or regulations set by governments, for the purpose of the current study, we treat it as a design parameter that allows us to explore the tradeoff between parking, fleet size and vehicle travel. If human mobility flows were perfectly balanced and evenly distributed throughout the day, no such tradeoff would occur—i.e., if for each passenger delivered at a destination, there were another passenger at the same location available for immediate pickup. In practice, however, there is overwhelming literature showing that human flows are highly unbalanced spatially and temporally^35,41–43^; thus, it is important to observe that, unless sharing of *rides* is considered, the sharing of *vehicles* cannot directly reduce the total traveled distance.

In Fig. 1 (right panel), each point represents one realization of our estimation with values of $r_{{\max }}$ ranging from $500\,\mathrm {m}$ to infinity. The first striking observation is that relative reductions in parking demand closely track relative reductions in fleet size. In fact, there is an almost constant ratio of $\sim 2$ between the two quantities (see SI Table S2). Intuitively, this is explained by the fact that each OV uses parking in two locations: close to residential areas where people start their commute in the morning, and in the city center where parking is necessary to be able to serve trips in succession (where moving to park in a remote location would take up too much time). Enforcing a strict separation between short-term waiting areas used for pick-up and drop-off and long-term parking can change this picture however, as we show in Supplementary Material (Sect. 3.3, SI Fig. S11). Nevertheless, such a strict separation will force OVs to travel even longer distances to find parking during the day and result in further increases in VKT, up to 35%, as shown in Fig. S11 in the Supplementary Material, where the number of parking spaces tends to 1, while an additional 0.4 “waiting space” is required on average per vehicle to handle passenger pick-ups and drop-offs. Additionally, as shown in Fig. S6 in the Supplementary Material, and in the discussion below, the number of pick-up and drop-off areas is highly dependent on the spatial discretization of trip start and end locations.

A second important observation is that the relationship between the extra distance traveled $TD_{extra}$ and the unified variable $N_{ov}=(NV_{ov},NP_{ov})$ can be empirically described by an exponential function

$$\begin{aligned} TD_{extra} = e^{-a NV_{ov}}, \end{aligned}$$

where $a = 9.6$ ($R^2 = 0.976$). This result implies a law of diminishing return for the control parameter $r_{{\max }}$. A short search radius of $500~\mathrm {m}$ is already sufficient to absorb many inefficiencies related to asymmetric and unbalanced mobility flows, achieving over 57% reductions in both fleet size and parking needs compared to the baseline scenario. At the same time, $TD_{extra}$ is limited to about 1.3%. On the other hand, if higher reductions in fleet size/parking infrastructure are sought, the search radius should be significantly increased, up to $5~\mathrm {km}$ or above. Despite the 10-fold increase in search radius, $N_{ov}$ is reduced by only 82% (2.45-fold reduction), while empty travel kilometers are increased to about 18% over $TD_{cur}$ (over 13-fold increase). This highlights that additional gains might not be worth the cost of additional VKT after some point and that cities and fleet operators might have very different interests in this regard, thus the outcomes will depend to a large extent on the regulatory environment and the tools local governments are given to influence fleet operations.

One way to offset additional VKT is to encourage sharing of rides. As we show in the Supplementary Material, Figs. S4 and S5, increase in VKT could be compensated if some of the trips were shared, with multiple passengers using the same vehicle. In accordance with our previous finding of the exponential nature of the tradeoff between parking and VKT, the ratio of trips that need to be shared to offset VKT increases is highly dependent on the $r_{{\max }}$ control parameter: only a few percent of trips need to be shared in the case of $r_{{\max }} = 500~\mathrm {m}$, while the ratio of shared trips need to be up to 30–50% to compensate VKT increases for the cases of $r_{{\max }} = 5~\mathrm {km}$ and $10~\mathrm {km}$. Given that the basis of our analysis is trips currently made in private vehicles, i.e. trips where convenience is likely an important factor, and also that the ratio of shared rides is typically lower than these values for ridesourcing operators^44,45^, it is questionable whether completely eliminating VKT increases with ridesharing will be feasible.

**Figure 2:**
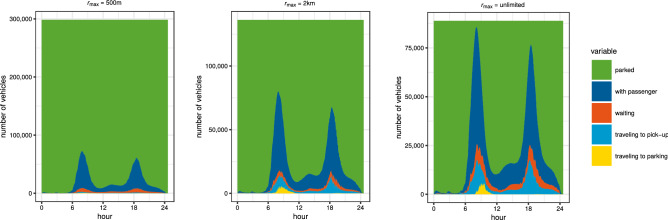
Utilization of fleet during the day for $r_{{\max }} = 500\,\mathrm {m}$ (left), $2\,\mathrm {km}$ (middle) and unlimited (right). The upper limit of the y-axis scale indicates the total fleet size necessary, ranging from $\sim$ 300,000 vehicles in the left panel to $\sim$ 100,000 in the right panel. Figures were created with the ggplot2 R package, version 3.3.0, available at https://ggplot2.tidyverse.org/, running on R version 3.6.3, available at https://www.r-project.org/. Additional editing was performed in Inkscape, version 0.92.3, available at https://inkscape.org/.

The significance of $r_{{\max }}$ is even more evident when looking at the utilization of the fleet during the day as displayed in Fig. 2. For small values of $r_{{\max }}$, fleet usage is limited by the ability of vehicles to reach trip requests. For large values of $r_{{\max }}$, fleet size is determined by the need to serve peak hour demand which is significantly higher than at other times.

### The role of waiting areas

The analysis so far considers only *parking* demand, i.e. storage for OVs that are not in service. In the following, we further investigate *pick-up* demand, i.e. space for OVs to wait while picking up a passenger. If users prefer short or zero wait times for an on-demand trip, the vehicle will need to (at least in expectation) arrive prior to a trip’s start time and wait in a suitable area. Ordering a vehicle in advance can ensure this, but can introduce an uncertainty in how long the vehicle needs to wait for the passenger. This is currently discouraged by operators by charging extra fees for vehicles waiting and presenting a user interface that emphasizes the immediate availability of vehicles. Especially with the adoption of AVs, the economics of this situation could change, with the cost of vehicles waiting decreasing, and operators realizing the value of receiving trip requests early that allows more optimized dispatching and the appeal of passengers not having to wait, essentially creating incentives to order a vehicle early even if the exact departure time is not known yet. Such vehicles will need to wait in a convenient location, that we term *waiting areas* in general and which could be interchangeable with long-term parking or be required to be separate from them.

**Figure 3:**
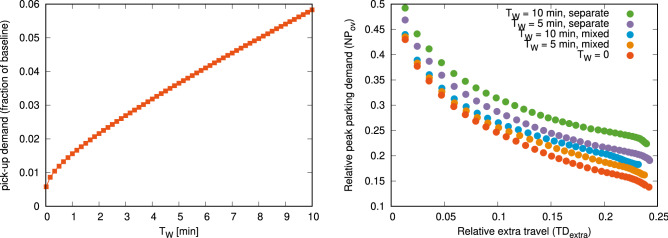
Estimating short-term parking requirements. Left: pick-up demand (i.e. parking spaces used as waiting areas) relative to the baseline parking demand as a function of the passenger convenience parameter ($T_W$). For $T_W = 10\,{\min}$, this number reaches almost 6% of the baseline (current parking demand), i.e. about 80,000 spaces. Note that this number is independent of the simulation parameter $r_{{\max }}$, since in all cases, waiting cars need to be present at the trip start nodes; compared to the long-term parking demand, this number will correspond to increasingly larger ratio as $r_{{\max }}$ is increased. Note that the curve starts from $T_W = 1\,\mathrm {s}$, where the pick-up demand is the sum of the maximum number of trips starting at the same time at each discrete location, i.e. about 8,000 parking spaces. Right: total parking demand ($NP_{ov}$) including pick-up demand as a function of increase in VKT ($TD_{extra}$) considering different expected extra required wait times ($T_W$) and usage policies for short-term parking. Note that these numbers also include the increased demand due to fleet size increases to compensate for the extra time spent idle. Results for $T_W = 0$ are the same as displayed in Fig. 1. Figures were created with Gnuplot, version 5.2, available at http://gnuplot.info.

We incorporate demand for such waiting areas in our model via the passenger “convenience” parameter $T_W$ and require vehicles to arrive at least $T_W$ time before the start of each trip, with the need to provide parking for them for this time. Using this assumption, we calculate the number of short-term waiting spots needed to accomodate this additional demand as the sum of the maximum number of waiting vehicles at each discrete location and interpret it as the “price of convenience”. As displayed in Fig. 3 (left panel), total demand for waiting areas can be as large as 6% of our current estimate of parking supply (for $T_W = 10\,{\min}$). Since this demand is assumed to be at the exact location passengers start their trips, it is also highly affected by how such locations are discretized in space—i.e., the extent to which pick-up locations are consolidated in a central point on each block or groups of blocks (see Fig. S6 in the Supplementary Material and the discussion in the “Methods” section for more details).

How much pick-up demand will add to the total parking demand of a city will depend on the policies related to use of short-term and long-term waiting areas, i.e. whether waiting areas can be used for long-term parking as well. We investigate two scenarios for this; in Fig. 3 we show that if there is a strict separation between short-term waiting areas and long-term parking (“separate case”), combined demand is significantly higher than if waiting areas are available for long-term parking as well (“mixed” case). The assumptions used in this estimation are further discussed in the Supplementary Material, Sect. 3, along with more detailed results for each case (Figs. S6–S10).

## Discussion

Our results show that a drastically reduced fleet of OVs could serve all private vehicle trips in Singapore, freeing up tremendous amount of space currently dedicated to parking. Parking lots could be redeveloped for housing and other productive uses, in turn stimulating the creation of dense, walkable neighborhoods and reducing urban sprawl and the associated societal and environmental costs^9,46^. In the minds of some planners, OVs and AVs have the potential to spur an evolution towards a more pedestrian-oriented society and make urban living more attractive^47,48^. At the same time, reducing the size of the vehicle fleet has been linked to the potential of reducing lifecycle emissions from vehicle manufacture and disposal^39,49^.

We do, however, identify four major limitations to the potential to reduce the amount of urban land devoted to parking.

First, the more urban land is freed up through parking consolidation, remote parking, and a shared fleet model, the more vehicle travel will result from deadheading and other vehicle relocation activity, e.g. vehicles moving to and from the remote lots. While parking demand can be reduced to as little as 14% of the baseline, this comes at the expense of a 24% increase in VKT.

Second, human mobility is temporally concentrated, particularly in the morning and afternoon peak commute periods^41–43^. Most vehicles will be idle at night, and many during the middle of the day, and require parking. While such parking facilities could be in remote locations, this would further increase vehicle travel from deadheading.

Third, there is a spatial mismatch between the places where reduced parking is most beneficial, and the places where OVs can most efficiently contribute to parking reductions (see Figs. S13–S16 in the Supplementary Material and “Supplementary Videos” for the spatial distribution of parking). Most of the freed-up spaces are in residential areas, while the most valuable land is in the central business district.

Fourth, pick-up demand can constitute a large amount of the total parking demand of an OV fleet, up to 25% in highly optimized scenarios (see SI Fig. S9). As these locations need to be easily accessible to passengers, their placement and design is less flexible and can thus hamper the efficient parking solutions provided in the future by autonomous vehicles. At the same time, we expect that the size of pick-up areas can be limited if we assume on-demand vehicles to be identical and thus interchangeable. Passengers could start their trip in any available vehicle which are replaced by cars from long-term parking areas.

In conclusion, our results show that very large swaths of urban land currently used for parking could be freed thanks to OD mobility. Furthermore, a range of trade-offs between parking reductions and increase in VKT have been quantified. While the equilibrium condition of a given city will depend on the actions of private fleet operators, governments have several tools to influence the latter’s behaviors. Deadheading can be discouraged by increasing the cost of vehicle travel through higher fuel taxes, congestion pricing or fleet-wide controls on vehicle utilization for operators^50,51^. Parking can be discouraged in central areas through parking taxes or mandatory price increases^10^ and land-use planning; at a minimum, cities can eliminate distortionary regulations that require a minimum amount of parking^4,7^, or establish maximums instead^52^. Planning ahead using data-driven decision-making—as enabled by approaches such as the one outlined in this paper—is especially important when considering future scenarios where shared AV fleets might make OD mobility the predominant mode of transport^36,37,53,54^.

## Methods

To estimate parking needs and travel of a fleet of OVs, we investigate the hypothetical scenario where every person currently using a private car for trips in the city–state of Singapore is willing to switch to using a shared mobility service; we are thus exploring solutions which serve all current trips made by private vehicles. We estimate such trips using SimMobility, an integrated agent-based simulation platform for urban mobility capable of giving realistic estimates of trips made by the target population and calibrated to represent Singapore in 2012^34^. Our methodology extends on our previous work focusing on a model of commuting^28^ and methods employed by Santi et al.^24,55^ with regards to ride-sharing and taxi fleet size estimation; now we focus on general trips for the whole population that are based on an extensive simulation of urban mobility in Singapore^34^. Our main goal is to estimate the number of vehicles $NV_{ov}$, parking spaces $NP_{ov}$ and extra travel $TD_{extra}$ needed to accommodate all trips made in private vehicles under ideal conditions. We then compare our results to the current number of cars, parking and travel distance, denoted by $NV_{cur}$, $NP_{cur}$, and $TD_{cur}$, respectively. Note that $TD_{cur}$ is defined as the sum of the travel distance of the *NT* given trips along shortest routes, under the assumption that there is always a parking spot available at the destination of a trip. Thus, $TD_{cur}$ can be considered as a baseline minimum distance that needs to be traveled to transport all passengers from respective origin to destinations. In this paper, we consider the variables representing OV system performance—$NV_{ov}$, $NP_{ov}$, and $TD_{extra}$—as normalized vs. the current system parameters. Formally:

$$\begin{aligned} NV_{ov}\triangleq \frac{NV_{ov}}{NV_{cur}}, NP_{ov}\triangleq \frac{NP_{ov}}{NP_{cur}}, TD_{extra}\triangleq \frac{TD_{extra}}{TD_{cur}}. \end{aligned}$$

Our approach for fleet size estimation is in contrast to most previous work, which analyzed the operational characteristics of autonomous mobility on demand (AMOD) services under pre-determined fleet sizes^1,21,22,26,35^; our main interest is providing lower bounds on fleet size, parking requirements and travel and explore the tradeoffs among these bounds. In this goal, our work is most similar to that of Spieser et al.^23^ and Vazifeh et al.^24^. The main difference from Ref.^23^ is that our approach also results in an idealized dispatching strategy which satisfies all trips without delay, while the estimate presented in^23^ is an absolute minimum that does not take into account operational characteristics, resulting in a need for a much larger fleet to provide adequate service to most passengers. In contrast, the work of Vazifeh et al.^24^ only focused on serving taxi trips and on ideal fleet size and did not consider parking or extra travel.

Our focus on parking demand is most similar to the aims of the previous study by Zhang and Guhathakurta^26^; the main difference again is that instead of running the simulation based on a presumed fleet size and parking availability, we aim to calculate the *minimum numbers* based on our constraints. Our work is complementary to recent work by Millard-Ball^30^, who present a detailed simulation of parking strategies on *privately-owned* AVs and to Xu et al.^27^ who investigate parking needs of ridesourcing vehicles. In contrast, we are explicitly interested in the scenario where the adoption of shared mobility brings along a shift in vehicle ownership as well.

### Trip data and estimate of current parking supply

The input for our analysis is a dataset of trips made by private vehicles generated by SimMobility, a complex platform for generating and simulating urban mobility realistically, based on a thorough process of calibration and verification using data including household travel surveys from the 2010 to 2015 period^34^. Our dataset focuses on Singapore which is currently the main target of SimMobility. The data includes 1.44 million private car trips made by 676 thousand individuals over the course of one day in the simulation. Trips have a median distance of $7.2\,\mathrm {km}$, average distance of $8.9\,\mathrm {km}$ with a standard deviation of $6.6\,\mathrm {km}$. The number of trips is realistic for Singapore, a city-state of about 5 million people with one of the lowest number of private vehicles per capita in the developed world, but still suffering from the effects of congestion in peak periods and dedicating significant resources and space to road infrastructure. The trip data is generated by SimMobility’s mid-term simulator module as trip chains taken by the agents in it, based on a calibrated model of present-day Singapore. Due to the nature of the modeling, this process results in one day’s data, that can be considered as a typical workday in Singapore. We are not using SimMobility’s capabilities to evaluate changes in mode share due the introduction of ride-hailing and AVs^35,37^; instead, we are assuming that every trip made in private vehicles today would be substituted with a ride in an OV, thus we can investigate what are the implications of such a setup under simplified conditions.

Besides trip data, SimMobility includes a database of buildings in Singapore^56,57^. We estimate current parking supply from this database and publicly available data from official sources: the minimum parking requirements published by the Land Transport Authority (LTA)^58^, the list of parking spaces managed by the Urban Redevelopment Authority (URA)^59^, and the aggregate number of parking spaces managed by the Housing Development Board (HDB)^60^. We further combine this data with results for parking occupancy from the trip data itself. This process results in an estimate of 1,369,576 parking spaces, or 2.03 parking spaces per person. We note that the real number is potentially even higher as our estimate is based on minimum requirements and the minimum amount needed to satisfy current trips made in private vehicles; in practice, developers may exceed the minimums. For comparison, parking supply for cities in the USA is estimated at between 2.49 and 3.3 spaces per vehicle^2,5,6^. We note there are significant policy and land use differences between Singapore and the USA, thus it is reasonable that the Singapore levels are lower. Furthermore, as of 2019, Singapore has started significantly reducing minimum parking requirements and imposing maximum limits on parking for new development^52^; nevertheless, since our simulations were calibrated based on data in the 2010–2015 period, it is reasonable that we use the prevailing minimum parking requirements at that time^58^. We describe the procedure for estimating the number of parking spaces in more detail in the Supplementary Material, in Sect. 4, with numbers of different types of parking displayed in Supplementary Table S4 and the spatial distribution of estimated parking supply in Fig. S13.

### Trip estimation based on bipartite matching and heuristics

Our main methodology for assigning vehicles to trips and parking is given in detail in the Supplementary Material as Algorithms 1 and 2. It proceeds by first separating trips into start and end “events” and then processing these events sequentially (“greedy heuristic”, Algorithm 1) or in batches (“bipartite matching”, Algorithm 2). In both cases, an event is considered to be successfully processed if either (1) the end of a trip is matched to the start of a consecutive trip such that the same vehicle is able to serve the later trip after finishing the earlier one; (2) the start of a trip is matched to a parked vehicle, that is available to serve it; (3) the end of a trip is matched to an unoccupied parking location, meaning that the vehicle will park there after finishing the trip. Events that are not matched are then satisfied by adding more cars and parking to the simulation, similarly to previous works^28,61^. This way, processing starts with zero cars and parking, creating only a minimal number of both over the course of processing all trips. We note that the parking spaces counted by this methodology are assumed to be used exclusively by OVs, but obviously, any specific parking space will be typically used by multiple vehicles during the day. This methodology also naturally allows us to keep track of vehicle utilization over the course of the simulation. We display the results of this in Fig. 2.

**Figure 4:**
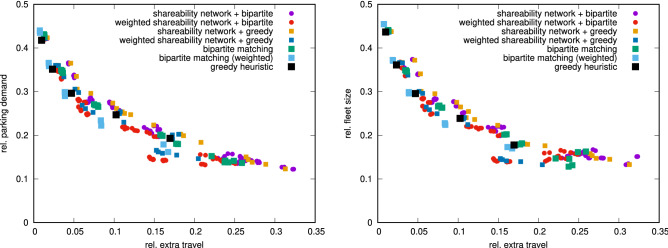
Comparison of results achieved with different approaches of assigning vehicles to trips and parking. Left: results for parking demand. Right: results for fleet sizes. For smaller fleet sizes, the approaches based on weighted matching tend to significantly outperform the other variations. Figures were created with Gnuplot, version 5.2, available at http://gnuplot.info.

The main results of this paper were obtained by the bipartite matching methodology presented as Algorithm 2 in the Supplementary Material. Comparison of different methodologies is presented in Fig. 4; results fit on the same trend as the main results displayed in Fig. 1. A further variation is that each maximum matching problem can be performed as a weighted maximum matching by weighting the edges in the graphs by the distance of the connection. In this way, the result will minimize the connection distances while maximizing the number of matches. This results in general better results (see Fig. 4); we note that this comes at the cost of longer runtimes. We note that the greedy heuristic estimate (Algorithm 1) is an extension of our previous work^28^, while the main idea of performing a matching among trips in batches is similar to the online solution for taxi dispatching given by Vazifeh et al.^24^ and the online matching problem studied in detail by Lowalekar et al.^62^; the main difference and thus a necessary extension is to consider parking for vehicles not in use at any point in time.

Beside matching trip requests in an online fashion or in batches, we also incorporated the global shareability network approach of Vazifeh et al.^24^. This corresponds to a model, where an “oracle” has knowledge of all trip demands in a day in advance and can decide on an optimal dispatching strategy based on that. We combined this approach with Algorithms 1 and 2 to be able to keep track of parking usage as well.

### Effect of discretization of space

The main results of the paper were obtained by considering a set of 4,529 discrete “nodes” that can be the start and end locations of trips, with parking possibly present at any of these nodes. Travel distances and travel times were estimated between all node pairs based on the real road network and travel data. This raises the question if this discretization can affect the result of the simulation. We note that the discretization is present in a real city as well as there are a discrete set of buildings and associated parking locations and garages that can act as trip origins and destinations. Nevertheless, using only 4,529 nodes is still an approximation. To test this, we also implemented a version of the main simulation in a continuous space model, where trips can start and end at any location, distances are taken as the Euclidean distance between points and travel times are calculated assuming a constant average travel speed. In this case, we adjusted the random start and end location of trips independently, by adding a variation to their coordinates chosen at uniformly random in an interval between $[-167\,\mathrm {m}, 167\,\mathrm {m}]$. We find that the results of this modified simulation agree well with the main results presented in this paper and increased fleet VKT can be modeled as an exponential function of the fleet size on an increased range of possible realizations. We display these results in the Supplementary Material in Figs. S2 and S3.

Discretization in space can further significantly affect results for the number of parking spaces used as waiting areas, since these need to be provided at the exact trip start locations. To account for this, we have carried out further tests, where trip start and end events were disaggregated randomly among $N_s$ discrete locations for each of the original nodes in our dataset. The results of this analysis show that the number of spaces used as such waiting areas is highly dependent on the choice of $N_s$, showing an approximate linear growth with increasing $N_s$ (i.e. increasing number of distinct location), as we display in Fig. S6 in the Supplementary Material. Even when only counting the absolute minimum required pick-up locations (the case of $T_W = 1\,\mathrm {s}$, i.e. “instant” pick-ups), these correspond to 3% of the baseline parking demand for $N_s = 10$ and over 10% of the baseline for $N_s = 40$, a case where each building in Singapore is assumed to have its own waiting area (see “Supplementary Material” for more explanation). This latter value is already comparable to the *total* long-term parking demand for the highly optimized cases among our results. Requiring longer pick-up or waiting times of cars for passengers (i.e. higher $T_W$ values) results in even higher demand for waiting areas.

This highlights that policies regarding the allocation of waiting and pick-up areas for on-demand mobility can affect total parking demand and urban space use. Some pick-up areas will indeed need to be provided reserved space, while in other cases, pick-ups could happen curbside, using traffic lanes, or via a more flexible use of road space. These questions will need to be addressed in more detail, using microscopic simulation of vehicles arriving, waiting and meeting passengers that is beyond the scope of the current work.

## Supplementary information

Supplementary Information.

Supplementary Videos.
