# Supplementary material for: Addressing the “minimum parking” problem for on-demand mobility
Source: Sci Rep. 2020 Oct 8;10:15885. doi: 10.1038/s41598-020-71867-1 (PMC7544901; doi:10.1038/s41598-020-71867-1)
Supplement: Supplementary file 1 — Supplementary Information. [file 41598_2020_71867_MOESM1_ESM.pdf]

Addressing the "minimum parking" problem for on-demand mobility

Supplementary Material

Dániel Kondor[1,*], Paolo Santi[2,3], Diem-Trinh Le[1],
Xiaohu Zhang[1,2], Adam Millard-Ball[4], Carlo Ratti[1,2]
[1]Singapore-MIT Alliance for Research and Technology, Singapore
[2]Senseable City Laboratory, MIT, Cambridge MA 02139 USA
[3]Istituto di Informatica e Telematica del CNR, Pisa, Italy
[4]University of California Santa Cruz, Santa Cruz CA, USA
* E-mail: dkondor@mit.edu

1 Main methodology

In this section, we give a more detailed description of the methodology used to estimate fleet size and parking requirements for the on-demand vehicle service (OV) based on simulated trip data. The input of the methodology is a list of trips that people currently are assumed to make in private cars; we're assuming every trip will need to be served by OVs in the future. This way, the methodology examines a hypothetical scenario to characterize the maximum potential gains from the adoption of OVs. We note that the methodology does not depend on whether the OVs are autonomous or have a driver. Depending on which variation of the methodology is used, it relies either on the full knowledge of the trips in advance (i.e. knowing all trips made in a day in advance) or at least partial advance knowledge, i.e. knowledge of upcoming trip requests in a given time window (5-30 minutes, depending on the simulation parameters). While the latter case can correspond to a system requiring advance reservation, it is unclear whether such a system would be popular with users, who are now used to near-instantaneous reservations offered by transportation network companies (TNCs) and taxi reservation systems as well. Furthermore, all of our methodologies rely on a central controller to assign vehicles to passengers and parking.

Two variants of the main methodology for assigning vehicles to trips and parking spaces are outlined in Algorithms 1 and 2 (notations used in both cases are further listed in Table S1 and further steps of Algorithm 2 are detailed in Algorithm 3). These can be further combined with the shareability networks approach to work on trip chains representing an ideal dispatching strategy instead of individual trips. Both variants work by assigning (1) trip ends to trip starts (i.e. a vehicle makes a connection among consecutive trips); (2) trip starts to free cars (i.e. vehicles are assigned to trips); (3) trip ends to free parking (free vehicles are assigned parking). This process is illustrated in Fig. S1. In both variants, when a trip start or end cannot be assigned a suitable free car or parking, a new car or parking space is "created". This way, the simulation starts with zero parking spaces and cars and only the minimum amount are added over the course of the simulation. The main difference between the two approaches is the way trips are processed: Algorithm 1 processes trips sequentially, always processing one trip start or end event at a time, assigning the closest available vehicle or parking (this way, we refer to it as a greedy heuristic). On the other hand, Algorithm 2 processes trips in batches by using sliding time window that is progressed over the set of trips, performing optimized bipartite matchings in each step.

To make realistic decisions about vehicle assignment, we calculate the travel times based on real-world data as well. In the case of Singapore, average travel times between a set of road intersections were provided based on real traffic data at different times of the day and week. There are a total of 11,789 intersections, providing good coverage of the area.

1.1 Preliminaries and notations

Table S1 describes the main notations used in both algorithms. In both cases, we start with a set of trips (T), where each trip ($t \in T$) is a tuple defined by start location, end location, start timestamp and end timestamp. In this analysis, we represent locations by a set of discrete *nodes*. Nodes can correspond to intersection, buildings, etc., and are defined by their coordinates and are matched to the road network. In the case of Singapore, we have a total of 4529 nodes. Travel times and distances (shortest path along the road network) among nodes are calculated in advance before running the algorithms described here. Our methodologies could easily accommodate more nodes or other representation of locations (e.g. working directly with coordinates). Start and end timestamps of trips are considered fixed (and known in advance), thus discounting any potential

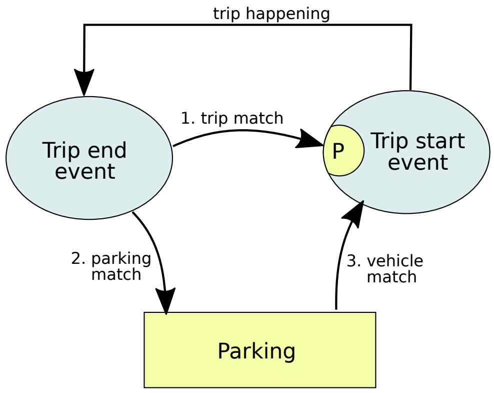

Figure S1: Left: illustration of the matching processes used to assign vehicles to trips and parking to vehicles. Figure was created with Inkscape, version 0.92.3, available at `https://inkscape.org/`

variations in traffic. This also means that cars have to be available exactly at the start time of all trips. Each trip is separated into a start and end event, which are stored in two separate lists of events (S and E respectively). Both algorithms then work on these event lists. For a start or end event ($s \in S$ or $e \in E$), we then denote their timestamp as t_s or t_e and their location as n_s or n_e. Since locations are represented as a discrete set of nodes, these can be directly compared for equality, while travel distances between locations are denoted by $d(e, s)$ and travel times by $t(e, s)$ and these are given by a lookup among the pre-computed values.

A main component of both algorithm are the list of available parking spaces and cars, denoted by L_P and L_C respectively. One parking space or car ($p \in L_P$ or $c \in L_C$) is represented by its location (n_p or n_c, from the same set of nodes that were used by the trips) and a timestamp (t_p or t_c) which is the time when the parking or car will become available. This is necessary since it will take some time for cars to reach parking or the start of the trips. E.g. if a trip finishes at t_e at node n_e, but a different node is selected for parking, the car will be only parked at time $t_c = t_e + t(e, c)$. When selecting the car to serve upcoming trips, this needs to be respected, i.e. it can only serve trip for which $t_s > t_c + t(c, s)$. Note that, similarly to the notation used for the distances and travel times between trip start and end events, $t(c, s)$ and $t(e, p)$ denote travel time for a car to reach the start of a trip from its current location or a car to reach the parking stop after finishing a trip. Similarly, $d(c, s)$ and $d(e, p)$ denotes the travel distances involved.

1.2 Greedy heuristic estimation

Algorithm 1 describes a greedy heuristic to assign cars to trips and parking spaces. It is a simple and efficient way for achieving this and calculating the minimum number of cars and parking spaces required by starting from zero available and recording the ones that were added during the course of processing all trips. The basic functioning is to process trip start and end events in sequence and always assigns the closest available car to a trip start and the closest available parking space to a trip end. Travel time to and from parking spaces is taken into account, i.e. it is recorded when a car reaches an assigned parking space and checked that it is able to reach the start of any new trip considered. A main parameter, r_{max} determines the maximum distance cars are allowed to travel to reach trip starts or parking. E.g. if $r_{\text{max}} = 1\,\text{km}$ then at the start of the trip, a car is only assigned if the closest available car is less than 1 km distance away. If no car or parking is available closer than $r_{\max}$ when needed, a new one is added to the system, increasing the count of total number of cars or parking (this happens in lines 41-43 and 54-55 in Algorithm 1). This way, we can start the simulation with zero cars and parking and only the minimum number will be added during the simulation.

A further consideration which is a separate part of Algorithm 1 is creating connections between consecutive trips. This means that instead of assigning a parking space after the end of a trip, a vehicle is assigned to serve an upcoming trip directly. This is an important part, since without this, a vehicle could be redirected to a parking space too far away, making it impossible to connect to an upcoming trip which happens closer or in a different direction. This is especially true for larger r_{max} values, where travel to more distant locations is possible. Connecting trips is faciliated by introducing a “look-ahead” parameter, t_{max}, and assuming that reservations for upcoming trips are made at least this time in advance. The value for this is determined as $t_{\text{max}} = r_{\text{max}}/\bar{v}$, where $\bar{v}$ is a lower bound for the average expected travel speed assumed for short relocation trip. In practice, we use $\bar{v} = 20\,\text{km/h}$. Note that since we are assuming that trip start times are fixed (are served without delay), we are essentially requiring reservations in advance of t_{max} even without trip connections, as this a bound on the time needed to reach a trip start from r_{max} distance away. Analysing a system with live

Algorithm 1 Greedy heuristic algorithm for determining fleet size and parking demand.

```
$T$ = { list of trips or trip chains }
$r_{\max}$ = maximum distance that OVs are allowed to travel empty
$t_{\max}$ = look-ahead time for trip reservations
$N_P = 0$ parking spaces required
$N_C = 0$ number of cars required
$D_{tot} = 0$ extra travel distance
$L_P$ = { empty list for free parking spaces (along with timestamps when they become available) }
$L_C$ = { empty list for available cars (along with timestamps when they become available) }
$S$ = { empty event list for start events }
$E$ = { empty event list for end events }
for all $t \in T$ do
    separate $t$ to start and end “events”
    add these to $S$ and $E$ respectively
end for
order $S$ and $E$ by timestamp
process events in time order with $t_{\max}$ shift between end and start events:
while $S$ and $E$ not empty do
    $s$ = first element in $S$
    $e$ = first element in $E$
    if $t_s < t_e + t_{\max}$ then                                   ▷ process start event
        $x = \arg\min_x d(x, s)$ where $x \in E$ s.t. $t_x \in [t_s - t_{\max}, t_s)$ and $d(x, s) < r_{\max}$ and
          $s$ is reachable from $x$ (i.e. $t_x + t(x, s) < t_s$)     ▷ find suitable matching end event
        if found (i.e. $x \neq \emptyset$) then
            remove $x$ from $E$
            reserve temporary parking at the location of $s$:
            if $\exists p \in L_P$, s.t. $n_p = n_s$ and $t_p < t_x + t(x, s)$ then
                remove $p$ from $L_P$
                add parking to $L_P$ with $s$’s location and timestamp
            else
                $N_P = N_P + 1$
                add new parking to $L_P$ with $s$’s location and timestamp
            end if
            $D_{tot} = D_{tot} + d(x, s)$                            ▷ keep track of extra travel of the fleet
        else                           ▷ no matching end event, find closest available parked car
            $c = \arg\min_c d(c, s)$ where $c \in L_C$ s.t. $d(c, s) < r_{\max}$ and can reach $s$ (i.e. $t_c + t(c, s) < t_s$)
            if found (i.e. $c \neq \emptyset$) then                  ▷ assign $c$ to the trip
                remove $c$ from $L_C$
                add $c$’s location to $L_P$ (with the timestamp: $t_s - t(c, s)$)
                $D_{tot} = D_{tot} + d(c, s)$
            else                                                     ▷ add new car and parking to the system
                $N_C = N_C + 1$
                $N_P = N_P + 1$
                add new parking to $L_P$ with $s$’s location and timestamp
            end if
        end if
        remove $s$ from $S$
    else                                                             ▷ process end event
        $p = \arg\min_p d(e, p)$ where $p \in L_P$ s.t. $d(p, e) < r_{\max}$ and is available (i.e. $t_p < t_e + t(e, p)$)
        if found (i.e. $p \neq \emptyset$) then                      ▷ assign $p$ as parking
            remove $p$ from $L_P$
            add $p$’s location to $L_C$ (with the timestamp $t_e + t(e, p)$)
            $D_{tot} = D_{tot} + d(e, p)$
        else                                                         ▷ add new parking to the system
            $N_P = N_P + 1$
            add car to $L_C$ with $e$’s location and timestamp
        end if
        remove $e$ from $E$
    end if
end while
```

notation	definition and usage
T<br>$t \in T$	list of trips to be served (or trip chains previously constructed)<br>one trip: each trip contains start and end locations and timestamps
S<br>$s \in S$	list of trip starts<br>one event in S, i.e. the start of one trip, represented by location and timestamp; we require that a vehicle should be available at this location and time
E<br>$e \in E$	list of trip ends<br>one event in E, i.e. the end of one trip, represented by location and timestamp; we require that the vehicle should be provided a parking location or assigned to a next trip
L_P<br>$p \in L_P$	list of available parking spaces (initially empty)<br>one available parking space; beside location, p also contains a timestamp, which is the time when the parking becomes available (i.e. this can be in the future for an already assigned trip)
L_C<br>$c \in L_C$	list of available cars (initially empty)<br>one available car; beside location, c also contains a timestamp, which is the time when the car becomes available
t_x (for $x \in S, E, L_P$ or L_C)	timestamp of entity (i.e. start or end time of a trip, or timestamp when a parking or car becomes available)
n_x (for $x \in S, E, L_P$ or L_C)	location of entity (start or end of trip or car or parking); locations are represented by one of discrete "nodes"
$d(x, y)$ (for $x, y \in S, E, L_P$ or L_C)	distance between the locations of two entities (e.g. the distance from an available car to the start location of a trip)
$t(x, y)$ (for $x, y \in S, E, L_P$ or L_C)	travel time between the locations of two entities (e.g. time it takes for an available car to reach the start location of a trip)

Table S1: Notation used in Algorithms 1 and 2.

reservations, especially with respect to passenger waiting times is beyond the scope of the current work and has been investigated in several different contexts already.

To be able to process connections between trips, we process trip ends and starts separated by a window of $t_{\max}$. This means that we process start events that are $t_{\max}$ into the future compared to the end events being processed: selecting the next start and end events s and e from the time ordered lists S and E, we select s to process if $t_s < t_e + t_{\max}$, and otherwise we select e (this is indicated in line 20 of Algorithm 1: processing of a trip start event happens in lines 21-46 and processing of a trip end event happens in lines 48-57). This way, when processing a trip start event, we not only look for cars which are parked, but also search among trip end events happening in the interval $[t_s - t_{\max}, t_s)$. If there are any trip end events in this interval which are suitable (meaning that they happen less than $r_{\max}$ distance away and the start event in question can be reached from them, i.e. $t_e + t(e, s) < t_s$), we select the one with the shortest travel distance as the match and assign its vehicle to serve the trip start event in question (this is done in lines 24-33 in Algorithm 1). In this case, the trip end event that was selected is not processed in the normal way to assign a parking to it (i.e. it is removed from the event list, so lines 48-57 are not executed for it). Nevertheless, even in this case, temporary parking is reserved at the start of the trip as the vehicle will typically arrive earlier than the start of the trip it is assigned to. For this purpose, only parking available at the start location (start node) of the trip is considered. If no such parking is available, the total number of parking spaces is again increased.

The result of this analysis is the total number of parking spaces needed after processing all trips (N_P), the total number of cars needed (i.e. fleet size, N_C) and the total "extra" distance traveled by the fleet (D_{tot}; this only includes distance traveled outside of making the trips themselves). Furthermore, the locations in L_P and L_C at the end of the simulation gives the locations where parking was needed during the simulation, thus our estimate of the spatial distribution of parking demand.

A further modification of Algorithm 1 is that it can be combined with an "ideal" dispatching strategy fonud by the methodology of *shareability networks*. In this case, first a dispatching strategy is generated by creating a bipartite graph among trip ends and starts and calculating a maximum matching on it; the result of this is a list of trip chains, each chain being a sequence of trips that are served by the same vehicle. In this case, we use these trip chains as the input instead of individual trips. This means that start and end events (S and E) will only include the start and end of whole trip chains instead of individual trips. In addition, since there could be need for temporary parking during a trip chain, we modify Algorithm 1 to process trip connections in the chain (supplied as separate input) in a similar manner to connections among trips found during running it, by

reserving temporary parking at the start of trips in a chain (lines 25-33).

1.3 Bipartite matching in batches

In contrast to Algorithm 1 which performs local optimization by considering one trip at a time, we present an alternative approach a Algorithm 2 which performs global optimization in batches, i.e. considering trips that fall into time windows a given size. The main idea is that trips (both starts and ends) in a window of T_w are considered and bipartite graphs are created from the possible connections (both among the trips themselves and among available cars and trip starts and trip ends and available parking). Then a maximum matching calculated on these bipartite graphs will result in a globally optimal solution for the assignment problem in that time window. Repeating the process with a sliding time window gives the solution for the whole set of trips considered.

In more detail, we create three bipartite graphs: (1) G_1, where edges connect trip end events to start events of later trips that can be reached from them; (2) G_2, where edges connect currently parked vehicles to upcoming trip start events that can be reached by them; (3) G_3, where edges connect trip end events to available parking spaces. Reachability is estimated using a dataset of estimated travel times between 11,789 intersections covering Singapore based on real traffic data including variation over a typical workday. The three graphs are created in three consecutive stages (G_1, G_2, G_3), each stage only including events that were not matched in the previous one: first, a matching is calculated among trip ends and starts in the time window under consideration (step **1** in Algorithm 2, lines 21-32). The result of this are direct connections among trips that only require temporary parking (handling the requirement for these temporary parking is done in step **6** in lines 85-95). Second, a matching among available cars and trip start events is performed (step **2**, lines 34-51). Third, a matching among trip ends and available parking spaces is performed (step **4**, lines 60-77). Trip start and end events which are not matched in any of the three stages are then satisfied by adding more cars and parking to the simulation, similarly to previous works [1, 2]. This way, processing starts with zero cars and parking, creating only a minimal number of both over the course of processing all trips.

A main parameter in these estimations is $r_{\max}$, the maximum distance cars are allowed to travel without a passenger (i.e. "empty distance"). When creating each G_i graph, only connections which include less than $r_{\max}$ travel are included; this way, we can limit the extra distance traveled by the fleet of OVs, although at the cost of more missed connections, thus requiring us to add more cars and parking. We repeat the processing with several different values of $r_{\max}$, ranging between 500 m and 10 km.

Further parameters affecting the result are the batch time window T_s and the look-out time window T_w. Among these, T_s is the time window in which trips are processed at a time, while T_w (with $T_w \geq T_s$) is the time window in which trips are considered. This means that matching is performed for all trips in the larger, T_w time window to result in a more optimal solution, while the solution is only accepted and processed in the smaller, T_s window. Trips which fall outside this smaller time window are processed in the next steps of the algorithm; after each step, time is advanced by T_s. A slight exception to this is matching trip start and end in steps **2** and **4**. In these cases, matching is performed twice: first for all events in T_w with processing the solution for those that fall in the smaller window T_s. In the second step, the same matching is performed only for any trips remaining in the smaller T_s window; the reason for this is to minimize adding more cars and parking spaces (i.e. prioritize serving events in the present instead of setting aside an excess amount of resources for events in the future). In practice, we ran the algorithm with several options for $r_{\max}$ between 500 m and 10 km. We used $T_w = 15$ min, while we explored the choices of 5 min and 10 min for T_s.

Algorithm 2 relies on several subroutines; among these, the functions for creating the bipartite graphs from a set of events are listed separately as Algorithm 3; furthermore, standard solutions are used for calculating matching on bipartite graphs such as the Hopcroft-Karp algorithm. A potential further addition is calculating weighted bipartite matching (i.e. minimum cost assignment problem) in each of steps **1**, **2** and **4**, using the connection distances as weights. This way, the solutions will attempt to minimize the extra travel taken by the fleet as well; we note that this version has significantly higher computational requirement, while resulting in somewhat better solutions.

1.4 Discrete and continuous space simulation

In the results displayed in the main text (Figs. 1 and 6), the above calculations were performed using a set of 4529 discreted locations ("nodes") as potential origins and destinations of trips. To better understand the relevance of this discretization, we also ran a modified simulation where each trip start and end event was assigned a random location independently in the neighborhood of the corresponding node (by adding a displacement to each coordinate, selected in a uniformly random way within $[-167\,\text{m}, 167\,\text{m}]$). Contrary to the main results, in this case, we assumed a simple Euclidean geometry, and a fixed average travel speed for OV operations that we varied between 10 km/h and 50 km/h. We were mainly interested in extending the simulation to smaller values of the $r_{\max}$ parameter, where the discretization can become especially relevant. While $r_{\max} < 500$ m is less

Algorithm 2 Stepwise matching algorithm for estimating fleet size and parking demand

$T = \{$ list of trips or trip chains $\}$
$r_{\max}$ = maximum distance that OVs are allowed to travel empty
T_s = time window for trips to process at a time
T_w = time window for trips to consider when performing matching ($T_w \geq T_s$)
$N_P = 0$ parking spaces required
$N_C = 0$ number of cars required
$D_{tot} = 0$ extra travel distance
$L_P = \{$ empty list for free parking spaces (along with timestamps when they become available) $\}$
$L_C = \{$ empty list for available cars (along with timestamps when they become available) $\}$
$S = \{$ empty event list for start events $\}$
$E = \{$ empty event list for end events $\}$
for all $t \in T$ **do**
  separate t to start and end "events"
  add these to S and E respectively
end for
order S and E by timestamp
$t = \min t_s : s \in S$ ▷ initialize first timestamp
while S and E not empty **do**
  process trips in $[t, t + T_s)$, while considering trips in $[t, t + T_w)$
  1. match trip ends to starts
    $G = \text{CreateGraphStart}(E, S, t, t + t_w)$
    $M = \text{BipartiteMatching}(G)$
    for all $(e, s) \in M$ **do** ▷ Process matched pairs ($s \in S$ and $e \in E$)
      if $t_e < t + T_s$ **then** ▷ If end event is in main time window, remove events matching
        remove e from E
        remove s from S
      else ▷ exclude e and s from the current step as a candidte matching
        mark e and s as reserved (will be used in next iteration again, but not in the following steps)
        remove (e, s) from M
      end if
    end for
    note: matches in M will be processed later
  2. match trip starts to existing cars
    $G = \text{CreateGraphStart}(L_C, S, t, t + T_w)$
    $M_S = \text{BipartiteMatching}(G)$
    for all $(c, s) \in M_S$ **do** ▷ Assign cars to trip starts ($c \in L_C$ and $s \in S$)
      if $t_s < t + T_s$ **then** ▷ Only process start events in the smaller time window
        remove c from L_C
        add parking to L_P with c's location and timestamp $t_s - t(c, s)$
        remove s from S
        $D_{tot} = D_{tot} + d(c, s)$
      end if
    end for
    $G = \text{CreateGraphStart}(L_C, S, t, t + T_s)$ ▷ re-do the matching in the shorter time window
    $M_S = \text{BipartiteMatching}(G)$ ▷ for the remaining start events
    for all $(c, s) \in M_S$ **do** ▷ ($c \in L_C$ and $s \in S$)
      remove c from L_C
      add parking to L_P with c's location and timestamp $t_s - t(c, s)$
      remove s from S
      $D_{tot} = D_{tot} + d(c, s)$
    end for
  3. process remaining start events (by adding more cars)
    for all $s \in S$ s.t. $t_s < t + T_s$ and s was not reserved in step #1 above **do**
      $N_C = N_C + 1$
      $N_P = N_P + 1$
      add parking to L_P with s's location and timestamp
      remove s from S
    end for

4. match trip ends to existing parking
  $G = \textsc{CreateGraphEnd}(E, L_P, t, t + T_w)$
  $M_E = \textsc{BipartiteMatching}(G)$
  for all $(e, p) \in M_E$ **do** ▷ Assign trip ends to parking ($e \in E$ and $p \in L_P$)
    if $t_e < t + T_s$ **then** ▷ Only process start events in the smaller time window
      remove p from L_P
      add car to L_C with p's location and timestamp $t_e + t(e, p)$
      remove e from E
      $D_{tot} = D_{tot} + d(e, p)$
    end if
  end for
  $G = \textsc{CreateGraphEnd}(E, L_P, t, t + T_w)$ ▷ re-do the matching in the shorter time window
  $M_E = \textsc{BipartiteMatching}(G)$ ▷ for the remaining end events
  for all $(e, p) \in M_E$ **do** ▷ ($e \in E$ and $p \in L_P$)
    remove p from L_P
    add car to L_C with p's location and timestamp $t_e + t(e, p)$
    remove e from E
    $D_{tot} = D_{tot} + d(e, p)$
  end for
5. process remaining end events (by adding more parking)
  for all $e \in E$ s.t. $t_e < t + T_s$ and e was not reserved in step #1 above **do**
    $N_P = N_P + 1$
    add car to L_C with e's location and timestamp
    remove e from E
  end for
6. process previously matched trips, provide temporary parking
  for all $(e, s) \in M$ **do**
    reserve parking at s's location
    if $\exists p \in L_P$ s.t. $n_p = n_s$ and $t_p < t_e + t(e, s)$ **then**
      remove p from L_P
      add parking back to L_P with s's location and timestamp
    else
      $N_P = N_P + 1$
      add new parking to L_P with s's location and timestamp
    end if
    $D_{tot} = D_{tot} + d(e, s)$
  end for
  $t = t + T_S$
end while

$r_{\max}$[km]	fleet size	parking requirements	ratio
current situation	676,227	1,369,576	2.03
0.5	297,674	588,768	1.98
1	234,384	476,511	2.03
2	178,099	367,536	2.06
5	121,196	247,621	2.04
1	98,802	194,421	1.97
unlimited	88,891	189,026	2.13

Table S2: Results for fleet size, parking requirements and the ratio of the two for the bipartite matching methodology (main results in the main text, i.e. Fig. 1).

Algorithm 3 Functions to create the bipartite graphs on for matching in Algorithm 2. For the sake of a simpler presentation, the logic of these function is shown as a nested loop, having a complexity of $O(|X| \times |Y|)$. When implementing these, we use a more efficient solution which employs an index based on travel times, thus the actual complexity is $O(|G|)$ (i.e. proportional to the number of edges returned).

function CreateGraphStart(X, Y, t_1, t_2)
    G = empty bipartite graph
    for all $x \in X$ s.t. $t_x \in [t_1, t_2)$ and x was not reserved in step #1 of Algorithm 2 **do**
        for all $y \in Y$ s.t. $t_y \in [t_1, t_2)$ and y was not reserved in step #2 of Algorithm 2 **do**
            if $t_x + t(x, y) < t_y$ **then**
                add (x, y) edge to G
            end if
        end for
    end for
    return G
end function
function CreateGraphEnd(X, Y, t_1, t_2)
    G = empty bipartite graph
    for all $x \in X$ s.t. $t_x \in [t_1, t_2)$ and x was not reserved in step #1 of Algorithm 2 **do**
        for all $y \in Y$ s.t. $t_y \in [t_1, t_2)$ and y was not reserved in step #2 of Algorithm 2 **do**
            if $t_x + t(x, y) > t_y$ **then**
                add (x, y) edge to G
            end if
        end for
    end for
    return G
end function

relevant from an operational viewpoint, extending the analysis to this regime allows us to better characterize the dependence among the resulting values of fleet size and additional VKT.

In Fig. S2, we compare results of this modified simulation using continuous geometry with the original results presented in the main text (as Fig. 1 – note that here the axes are reversed). We display the original simulation results as the "discrete" case (red points), along with the modified simulation results (for an assumed average speed of 50 km/h) as the "continuous" case (blue points). Furthermore, we display results with weighted matching in both cases as well (yellow and purple points). We also display an exponential fit to the results of the continuous simulation as black lines. This exponential form fits the results for the continuous space simulation quite well for the most of the range of results. It also fits the results of the discrete case nicely for $r_{\max} \geq 500\,\mathrm{m}$. We speculate that the deviation of the discrete case results for smaller $r_{\max}$ (larger fleet size and smaller extra VKT) is an effect of discretization: for small r_{max} values, the only possible connection will be between trips ending and starting at the same node for an increasing share of cases.

In Fig. S3, we compare results for different values of the assumed average travel speed in the continuous space case. We see that these results are very similar, with a "saturation" effect present for small fleet sizes (large r_{max}).

1.5 Vehicle shareability networks

The vehicle shareability network provides a computationally efficient way to calculate an optimal dispatching strategy for a given list of trips, resulting in minimum fleet size or minimum total connection distances between trips [3]. In this approach, a network is constructed from the trips where each trip is a node, and two trips are connected by a (directed) edge if the start of the later trip can be reached from the end of the earlier one. This way, each edge represents a trip pair that can be served consecutively by the same vehicle. Each trip can have several incoming and outgoing edges as there can be several different options of which connections to choose at the end of a trip. Notably, trips separated by large time intervals can always be connected by an edge; this way, the shareability network can be huge, scaling with the square of the number of trips. We limit the network size and computational complexity by setting a maximum threshold on the connection time as well. Since we expect that a good dispatching strategy will prefer short connections, reasonable values of maximum connection time will only slightly affect the solution. Having constructed this shareability network, we can find an optimal dispatching strategy by finding a minimum path cover on it. Since the shareability network is a directed acyclic graph, finding a minimum path cover can be achieved efficiently (in polynomial time) by converting the problem into finding a maximum matching in a bipartite graph [4, 5, 3]. This minimum path cover can then be used as a dispatching strategy where each path is interpreted as a chain of consecutive trips

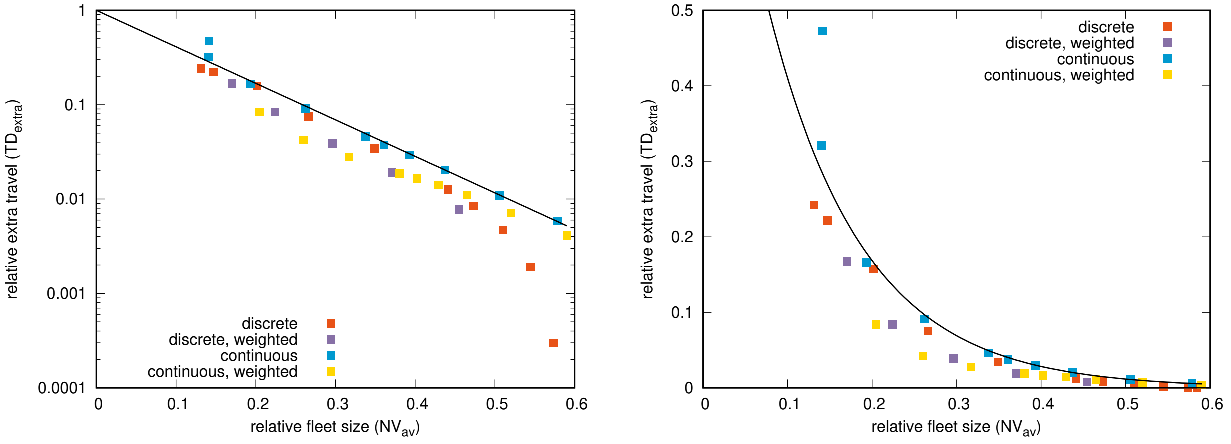

Figure S2: Total extra travel for the fleet as a function of fleet size for the discrete case (original simulations) and the simplified, continuous case (Euclidean space). Results are displayed for $r_{\max}$ values between 50 m and 10 km. The black line displays an exponential fit to the continuous case. The two panels display the same data with logarithmic and linear y-axis on the left and right respectively. Figures were created with Gnuplot, version 5.2, available at `http://gnuplot.info`.

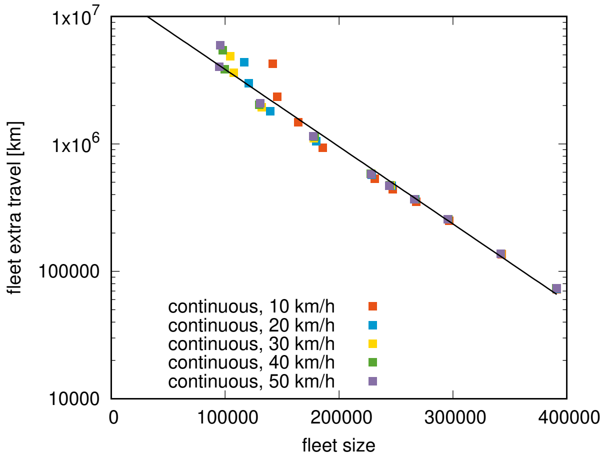

Figure S3: Total extra travel for the fleet as a function of fleet size for the continuous case, comparing results for different assumptions of average travel speed. We see that in this model, the average travel speed affects the results very little; there is a "saturation" effect limiting the minimum achievable fleet size as slower vehicle travel means less possible connections. Figure was created with Gnuplot, version 5.2, available at `http://gnuplot.info`.

$r_{\max}$ [km]	median [km]	average [km]	standard deviation [km]
0.5	0	0.083	0.153
1	0	240	342
2	0.098	0.574	0.712
5	0.565	1.382	1.66
10	0.691	1.911	2.576
unlimited	0.712	1.945	2.727

Table S3: Statistics of connecting trips for different values of $r_{\max}$. Typical trip lengths are singificantly below the allowed maximum.

to be served by the same vehicle. Thus, the number of vehicles needed is at most the number of paths found. We note that constructing the shareability network is equivalent to considering the G_1 graph in the previous method, but including all trips during the day (instead of only those in a limited time window T_w) and further limiting connection time instead of connection distance; this will result in some variation during the day due to variations in traffic speed.

Having identified trip chains in an ideal dispatching strategy, we still need to assign parking to vehicles at the start and end of chains and inbetween consecutive trips. Furthermore, since demand fluctuates during the day, some trip chains can be distinct in time, thus possible to serve with the same vehicle (if separated by more than the minimum connection time). For these reasons, we continue by using the trip chains as the input of any of the previously described methods ("bipartite matching" or "greedy heuristic", i.e. Algorithm 2 or 1) to arrive at a final dispatching strategy that includes assignment of parking as well.

While the result of this computation will be optimal in terms of fleet size, it can result in excessive extra travel as connection distance between consecutive trips in a chain is not part of the optimization. This problem is slightly mitigated by allowing only relatively short connection times, thus limiting connection distances as well. Furthermore, we can set an explicit limit on connection distances as well. An other approach that we implemented is again performing a weighted maximum matching after converting on the trip shareability network, giving a solution which minimized total connection distance as well.

Looking at the results in Fig. 6 in the main text, we see that the results of this approach are mostly comparable with the previous ones; nevertheless, the combination of weighted shareability networks with the bipartite matching approach is able to significantly outperform all the other methods, resulting in 85% reduction in fleet size and parking demand at a cost of only 15% extra VKT as opposed to the results obtained by performing only unweighted bipartite matching, which achieves similar reductions at the cost of 25% extra VKT. This outlines that performance in realistic conditions will be limited by operational constraints, i.e. the fact that trip requests are generally not known in advance as required to construct shareability networks for the whole day and perform a global optimization.

1.6 Actual length of connecting trips

While $r_{\max}$ limits the length of connecting trips (either between consecutive trips or between trip start or end points and parking), this is only the maximum possible value; actual distances traveled for individual trips will likely be shorter. We display statistics about these in more detail in Table S3.

2 Incorporating ride sharing

A potential way to compensate for the extra travel of an AV fleet is to encourage people to share rides. We use the methodology of Santi et al. [6] to estimate the potential for shared rides under the assumption that x percentage of people would be willing participate, with x varying between 0% and 100%. Not surprisingly, we find that a large percentage of trips are shareable. We display the shareability ratio as a function of people willing to share (similarly to Ref. [7]) in Fig. S4. We see that if everyone was willing to share, over 97% of all trips could be actually shared. Even if only 10% of trips passengers are willing to share, the shareability ratio is still above 70%. For each case, we then produce merged trips that represent serving two original trips wherever sharing is possible. We then repeat our analysis for this set of combined trips (i.e. merged trips and original trips that cannot be shared or passengers are unwilling to share) and display the results as a function of x and $r_{\max}$ in Figure S5. We see that as $r_{\max}$ gets larger (and dispatching gets more efficient in general), a larger x share of shared rides are required to compensate for added VKT of a shared fleet.

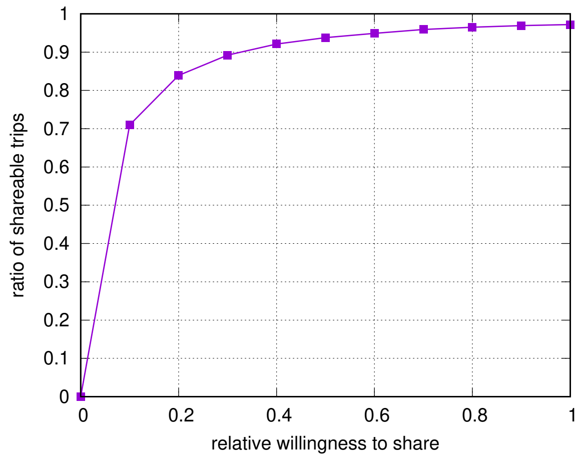

Figure S4: Shareability potential of trips. Ratio of trips actually shareable as a function of the ratio of trips where passengers would be willing to share their ride. Shareability ratio is relative to the trips available for sharing. Figure was created with Gnuplot, version 5.2, available at `http://gnuplot.info`.

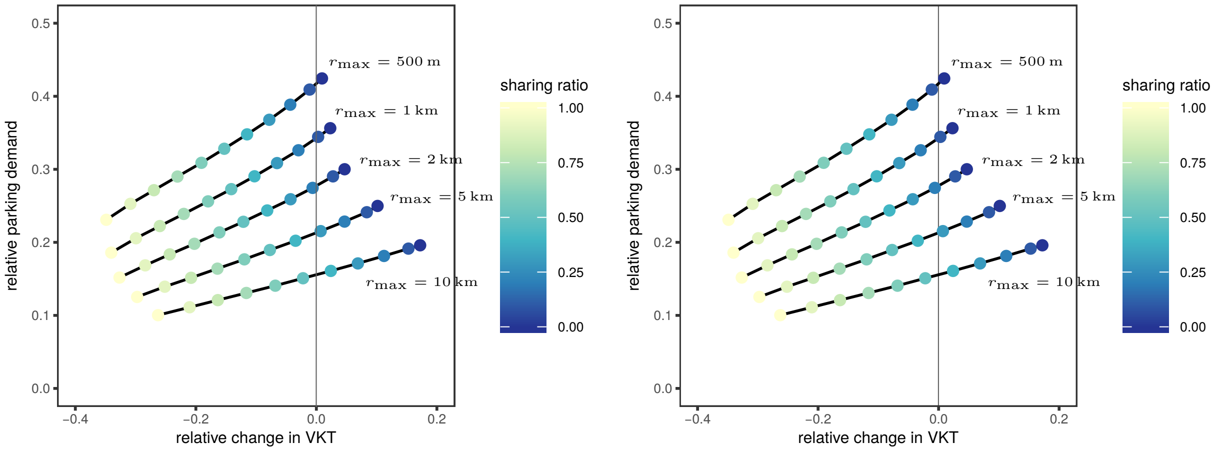

Figure S5: Results for fleet size, parking demand and change in total travel if people were willing to share rides. Color corresponds to the fraction of trips where people are willing to share rides (in 10% increments from 0% to 100%); lines correspond to $r_{max} = 500$ m, 1 km, 2 km, 5 km and 10 km from top to bottom respectively. Figures were created with the `ggplot2` R package, version 3.3.0, available at `https://ggplot2.tidyverse.org/`, running on R version 3.6.3, available at `https://www.r-project.org/`.

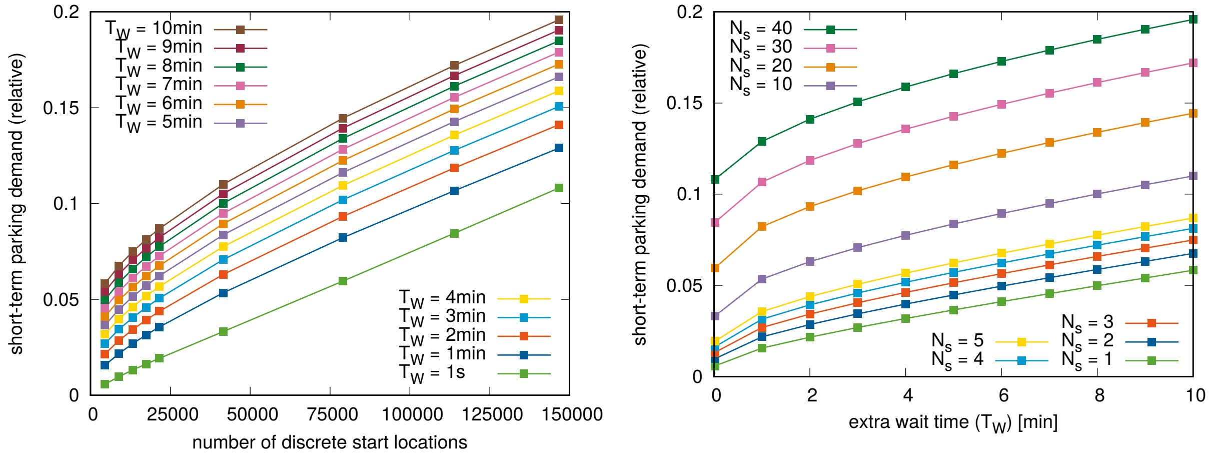

Figure S6: Estimation of pick-up demand (fraction of baseline parking demand). Left: pick-up demand for different choices of T_W as a function of the number of discrete nodes considered as possible trip start locations. Right: same results displayed as the function of T_W with distinct curves corresponding to different N_s parameter values and thus different number of distince nodes. Values displayed in this figure are the average of 100 realizations of the random process described in the text. Standard deviation of values among the 100 realizations is less than 0.25% in all cases. Figures were created with Gnuplot, version 5.2, available at `http://gnuplot.info`.

3 Estimating short-term parking requirements

Beside a change in the total number of parking, a further important consideration is a change in the use of parking on a local level. If people are willing to change from using private cars to OVs, actual parking facilities will be less important from the users' point of view (i.e. they will not need to be easily accessible for average users), while the provision and design of adequate pick-up, drop-off and waiting locations will become increasingly important. Furthermore, in a future scenario with autonomous vehicles (AVs) we expect users' expectations to change regarding the start of their trips: since the cost of waiting can decrease significantly as it no longer includes a driver's salary, users might prefer to order a vehicle in advance and have the vehicle wait for them instead of having to wait themselves. While this increases convenience for passengers, booking trips some time in advance can be beneficial for operators as well, since this will allow the use of a dispatching algorithm that aggregates requests in time, allowing more efficient solutions. This way, operators might change their pricing structure to incentivize this behavior as well. Vehicles waiting for passengers in pick-up locations could add a non-negligible amount of demand for temporary parking spaces.

In the following, we use the general term *waiting area* for parking spaces that need to be in locations easily accessed by passengers. We envision that these locations will include cars waiting for passengers who have not arrived yet, and also facilitate actual pick-ups and drop-offs. The actual spatial layout of such areas is an important research question by itself, a detailed investigation of which is beyond the scope of the current work. E.g. in the case of dense downtown locations, each building might provide such an area at its entrance (as is often typical for hotels or some office buildings nowadays), or the city could provide these in a manner similar to taxi stands. Some of these waiting areas could also be provided in parking garages, in locations that are most conveniently accessible on foot. In areas with less traffic, a waiting area might serve multiple buildings together, or waiting areas might be curbside, either in place of current curb parking or part of a traffic lane. Nevertheless, in the following, we assume that waiting areas are made up of discrete "parking spaces" as well, and need to be counted in totality. Also, in line with the rest of the analysis, we note that trips can start and end at a set of discrete "nodes"; we then assume that each node has a certain number of waiting spots that can only be used for trips that start at that specific node.

To estimate of the number of waiting spots needed, we then consider that vehicles have to arrive exactly T_W time before the start of each trip. We vary the T_W parameter between 1 s and 10 min. Low values of T_W then show the absolute minimum needed to handle pick-ups, while larger values account for presumed uncertainity in trip start times.

3.1 Absolute demand for short-term parking

Formally, for a trip t that start at node n_s at time t_s, we require the car serving it to be parked at the waiting area at n_s in the interval $[t_s - T_W; t_s]$. Based only on this consideration, we can calculate the number of waiting

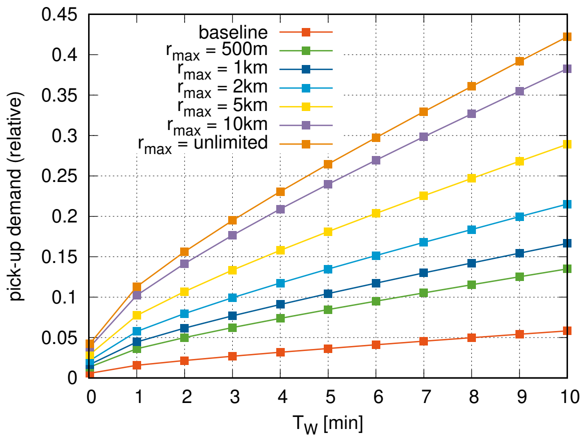

Figure S7: Pick-up demand (i.e. parking spaces used for short-term waiting) relative to the parking demand estimated previously as a function of the assumed extra wait time (T_W). Absolute numbers are the same for each curve, reaching about 80,000 parking spaces for $T_W = 10\,\text{min}$, but these correspond to larger and larger relative share. The curves start from $T_W = 1\,\text{s}$, where the pick-up demand is the sum of the maximum number of trips starting at the same time at each discrete location, i.e. about 8,000 parking spaces. Figure was created with Gnuplot, version 5.2, available at `http://gnuplot.info`.

cars at each node at any time as

$$N_W(n,t) \equiv \sum_s \delta_1\left(\frac{t - t_s + T_W}{T_W}\right)\delta_{n_s,n}$$

where the summation in s includes all trip start events (refer to Table S1 for notations), δ_1 is a 1-width Dirac-delta function, while $\delta_{n_s,n}$ is the usual Kronecker delta symbol, defined as:

$$\delta_1(x) = \begin{cases} 1 & \text{if } x \in [0,1] \\ 0 & \text{otherwise} \end{cases} \qquad \delta_{x,y} = \begin{cases} 1 & \text{if } x = y \\ 0 & \text{otherwise} \end{cases}$$

Using these, we can formally calculate the total demand for waiting areas as

$$N_W^{\text{total}} = \sum_{n \in \text{nodes}} \max_t N_W(n,t)$$

where the summation is done over all discrete nodes in the simulation. The result of this calculation is displayed in Fig. 3 (left panel) in the main text.

Since we calculate the maximum number of waiting cars per nodes (i.e. discrete locations), the discretization of space can affect the results for N_W^{total}. Clearly, if all trips started in distinct locations, the number of parking places for waiting cars would equal to the number of trips (1.44 million in our case). Having multiple trips start at the same discrete locations (nodes) allows them to share the same waiting area if their waiting times do not overlap. This way, using only 4529 discrete nodes as trip start and end locations can underestimate pick-up demand. To overcome this limitation, we repeated the previous estimation after splitting each node into N_s virtual locations and assigning trips to start at any of these at uniformly random. In Fig. S6 we display pick-up demand (N_W^{total}) estimated in these scenarios as a function of the total number of discrete nodes with at least one trip starting there (we note that the number of actual nodes is not necessarily $N_s \times 4529$ since some nodes have only a few trips starting there, so they do not generate N_s separate nodes). While it is unclear how many discrete pick-up locations will be needed in the future, a reasonable upper bound is the total number of buildings in Singapore. Exploiting the fact that in Singapore, every building is assigned a unique postal code, we can estimate the total number of buildings based on a database of postal codes. We downloaded the list of unique post codes from the API of OneMap.sg [8] using the scripts published at [9], finding a total of 121,363 unique postal codes. This way, we estimate that the upper end of the results presented in Fig. S6 show a realistic upper bound on the pick-up demand.

3.2 Combined estimate of total parking demand

While our previous analysis gives an estimate of pick-up demand by itself, we are also interested what is the combined effect on parking demand in our estimate based on trips. To do this estimation, we run a modified

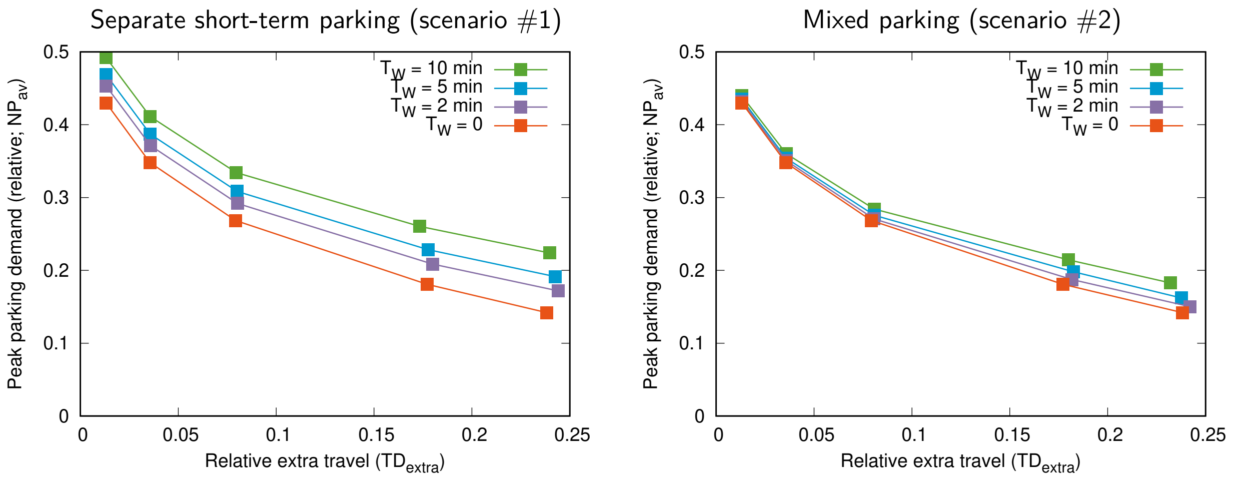

Figure S8: Estimating parking requirements including pick-up demand. Left: scenario #1, i.e. short-term and long-term parking are stricly separated. Right: scenario #2, i.e. pick-up areas can be used for long-term parking as well. Figures were created with Gnuplot, version 5.2, available at `http://gnuplot.info`.

version of the main estimation (Algorithm 2) that takes into account the requirement for vehicles to wait for passengers before the start of trips. We evaluate two distinct scenarios regarding the use of waiting areas:

1. Each location has a separate waiting area (i.e. pick-up locations) beside a normal facility for long-term parking (e.g. a parking garage). All vehicles that are waiting to pick up a passenger are required to park in the waiting area, while vehicles are not allowed to park there long-term. The size of this area is determined over the course of the simulation, i.e. we do not constrain it, but record the highest number of vehicles in the waiting area of each node at any time.

2. Instead of reserving a separate area only for vehicles waiting for a passenger, waiting areas can be used for long-term parking as well. We further assume that all vehicles are essentially interchangeable, thus passengers need not be assigned any specific vehicle, but will be able to start their trip in any of the vehicles available in the waiting area. This way, we do not have to explicitly divide parking into short-term and long-term facilities during the course of the simulation, but assume that vehicles always occupy the waiting area first and only park in the long-term facilities if the waiting area is full. For each discrete location, we record the maximum number of cars simultaneously waiting for passengers as the estimated demand for short-term parking. In this case, we can expect more optimal solutions in terms of combined (short-term and long-term) parking demand.

We note that the actual pick-up demand (parking spaces needed to accommodate waiting vehicles) will be the same in both scenarios, as the number of vehicles waiting at each node is only determined by the sequence of trips starting there and the T_W parameter, as estimated before. In the first scenario, this is added to the long-term parking demand, while in the second scenario, there can be an overlap, thus the total increase will be potentially less than the total need for short-term parking. In both cases, there is a potential increase in fleet size since vehicles need to spend some of their time waiting; in turn, this can add to long-term parking requirements as well.

Again, we vary T_W between 0 and 10 minutes, with $T_W = 0$ representing the original analysis without pick-up demand. Here in Fig. S8 we present the comparison among the two scenarios, while in Fig. S9, we show the relative share of short-term parking in scenario and the absolute number of short-term parking spaces. In Fig. S10, we display the change in fleet size in these cases.

Not surprisingly, scenario #1 results in higher increases in total parking needs as there parking designated as short-term cannot be reused for long-term parking purposes. In either case, our results emphasize the importance of designing pick-up and short-term waiting areas in an efficient way.

3.3 Effect of short-term parking policy of parking ratio

As illustrated in Fig. S1, the processes used to assign vehicles to trips and parking (Algorithm 2) has three steps where maximum matching is performed to optimize utilization of vehicles and parking. First, the end of trips are matched directly to start of trips (#1); this step will ensure maximum utilization for existing vehicles. Since a vehicle assigned to a new trip might arrive too early, parking is always reserved at the start node of the new trip, even if we are not counting short-term parking separately. After this step, the remaining trip end

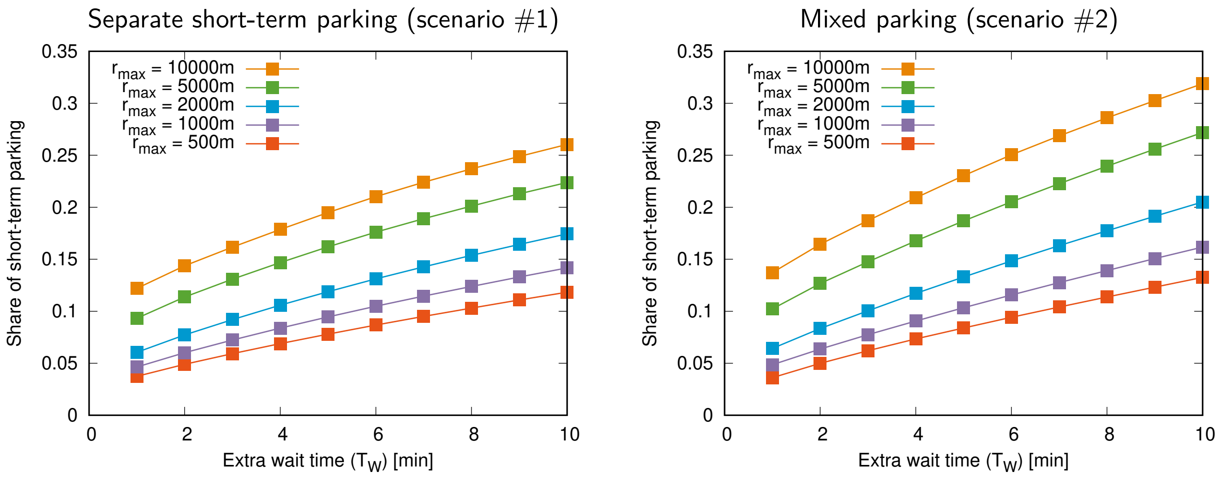

Figure S9: Relative share of pick-up demand (parking spaces used at least once for short-term wait) among the *combined* parking demand in both scenarios as a function of the extra wait time (T_W). Contrary to Fig. 5 in the main text where the pick-up demand was compared to the long-term parking demand without considering short-term waiting at all, here we display the share of parking spaces used for short-term waiting after running the modified simulation that takes into account extra wait as well. Figures were created with Gnuplot, version 5.2, available at `http://gnuplot.info`.

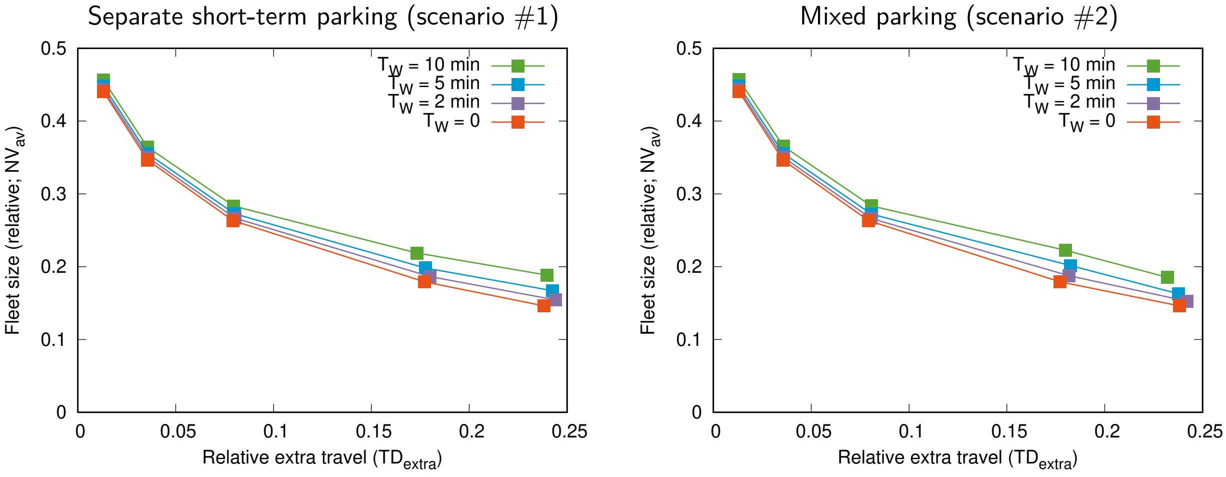

Figure S10: Change in fleet size requirement due to extra wait before the start of trips. Figures were created with Gnuplot, version 5.2, available at `http://gnuplot.info`.

and start events are matched to available parking and idle vehicles and respectively (#2 and #3). Remaining trip end and start events are then satisfied by creating new vehicles and parking. Including the first step (trip matching) can help in finding more optimal solutions, since after finishing a trip, vehicles need not travel to parking in potentially distant locations, but can serve any new trip request close by.

We explored multiple options regarding how parking is accounted for. In the original estimation, parking is not differentiated between the first process (trip matching, which results in essentially short-term parking demand) and the second and third one (which matched trips to long-term parking). Thus, the first process will result in parking created at nodes where trips typically start (and are matched to previous trips ending in the neighborhood). These parking spaces can then be used for long-term parking as well in later steps of the estimation. Results from this option are presented as "original results" in Figs. S11 and S12 and have a fairly constant ratio of parking to vehicles regardless of $r_{\max}$.

The orignal method (Algorithm 2) can be easily modified to not include the possibility for trip connections (case #1). In this case, whenever a trip is finished, the car serving it needs to find (long-term) parking, while new trip can only be served by cars that are idle (parked). This removes the requirement that parking is available at the trip start nodes. At the same time, this can result in unrealistic dispatching, especially if cars travel to far away parking lots instead of serving passengers closer by. Also, this case has the potential of underestimating parking, since all short-term parking requirements are neglected. This case is presented in Figs. S11 and S12 as the solution "without trip connections". In this case, the ratio of parking and cars starts out as constant as well and then starts to decline above $r_{\max} = 2\,\text{km}$, reaching almost one parking per car already at $r_{\max} = 10\,\text{km}$. This comes at the expense of further increase in VKT (between 10%-15% more than in the previous case). The tradeoff between parking and VKT however stays on the same trend as in the previous case (see Fig. S12).

An alternate way to manage parking demand is to explicitely separate parking into short-term and long-term categories as we did in the analysis considering extra wait times at the start of trips ($T_W > 0$ cases). Here, we set $T_W = 0$ (i.e. cars normally do not have to wait), but require that parking that happens when connecting trips is accounted as short-term parking and is completely separted from long-term parking (i.e. cannot be used for long-term parking). This is presented as the "$T_W = 0$" case in Figs. S11 and S12, with separate plots showing only the demand for long-term parking and the combined parking demand. If we only consider long-term parking, the ratio of parking to cars again approaches 1 (but only in the $r_{\max}$ = unlimited case). If we consider combined parking demand, the ratio stays above 2 up to $r_{\max} = 5\,\text{km}$ and starts to decrease after. Even for $r_{\max} = 10\,\text{km}$, it is still just around 1.7, indicating that decreasing the ratio of parking to cars below 2 while realistically accounting for parking can only happen with high $r_{\max}$ search radius, i.e. at the expense of large increases in VKT.

We emphasize that in all cases, despite the further decrease in parking demand and increase in VKT, the relationship between these two quantities is still well explained by the exponential form found in the main text of the paper (shown as a black line in Fig. S12).

4 Estimating current parking supply

We base the estimation of the current parking supply on minimum parking requirements and the need to find parking for each trip in the trip dataset we use. In more details, we arrived at this estimate of current parking supply with the following procedure: for housing constructed by the Housing Development Board of Singapore (HDB), we distribute the total number of parking reported officially evenly among all such apartments in Singapore. The aggregate number available from the government source [10] is 640,188, while the SimMobility database includes 1,188,649 HDB housing units in total, giving on average 0.5386 parking spaces per unit. We note that parking managed by the HDB is often used for multiple purposes, especially for retail as some of these are strategically positioned in local town centers and offer access to the general public. For other types of development, we use the minimum parking requirements published by the Land Transport Authority (LTA) that was valid for the period before February 2019 [11]. We note that LTA imposed significant changes from February 2019, lowering minimum requirements and establishing limits on maximum allowed parking for new developments [12]. Since the buildings considered in this work were completed before this date, we used the older standard. Based on this, for private housing (i.e. housing not constructed by the HDB), we assume one parking space per unit, giving a total of 321,974. Beside housing, we have four use categories for buildings in the SimMobility database: office, retail, factory and other with aggregate floor areas of 7.2, 5.2, 34.9 and 23.6 million square meters respectively. For these, we use minimum parking requirements that are most appropriate, resulting in a total of 22,458 parking spaces related to office use, 24,737 parking spaces related to retail, 77,577 parking spaces related to factory use and 78,829 parking spaces for other uses. Furthermore, we have 25,740 parking spaces managed by the Urban Redevelopment Authority (URA), mostly on-street parking and some parking lots [13]. Adding these up, we have a total of 1,191,503 parking spaces, giving a ratio of 1.762 parking spaces per person for our simulated population. We display a summary of different types of parking Table S4.

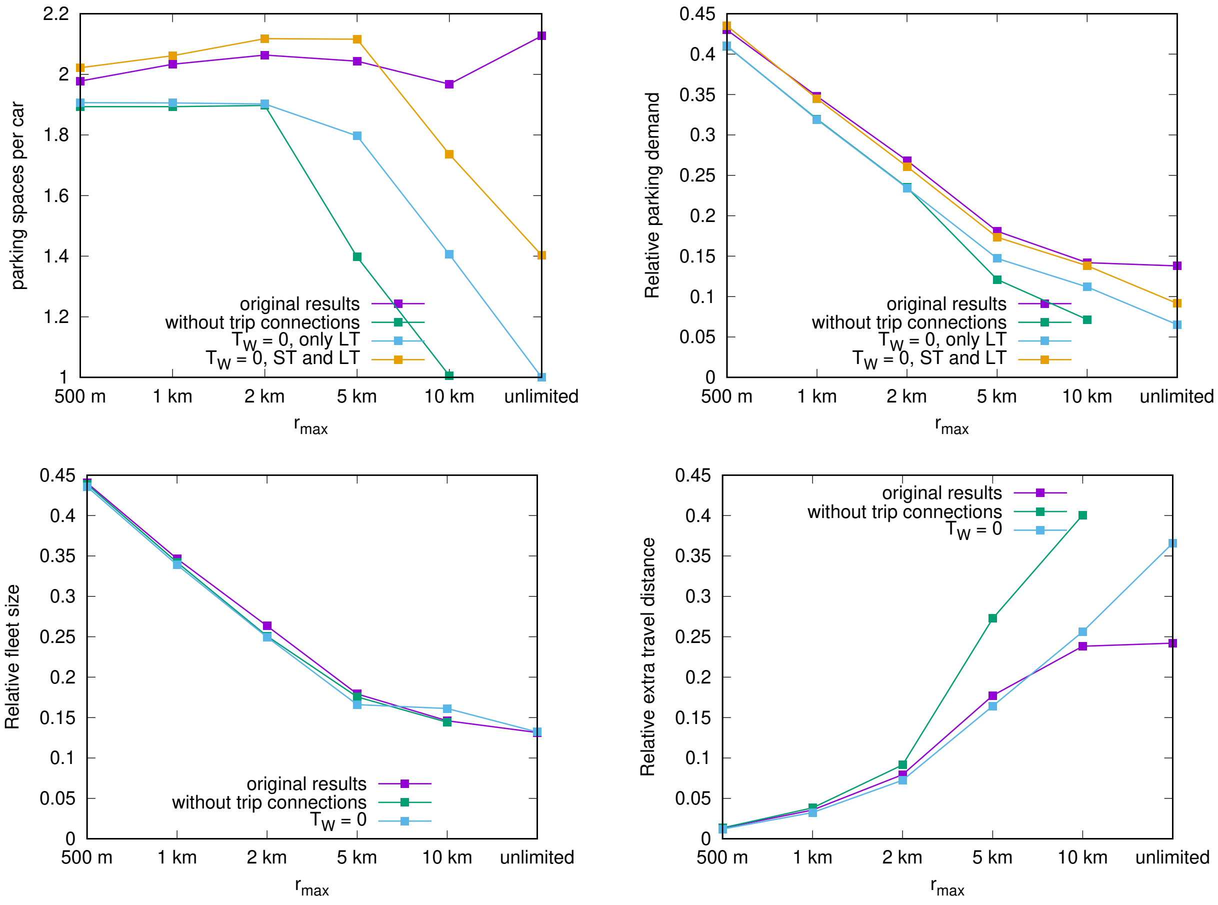

Figure S11: Comparing different methods to estimate demand for parking and fleet size. Ratio of parking to cars (top left), relative number of parking spaces (top right), relative fleet size (bottom left), and relative extra VKT (bottom right) are displayed as a function of $r_{\max}$. Among the cases presented here, "original results" refers to the bipartite graph approach presented as the main results in the paper (Fig. 1 and "bipartite matching" in Fig. 4); "without trip connections" refers to the same methodology, but omitting direct matching between consecutive trips (cars always have to return to parking at the end of a trip, new trips can only be matched to cars that are already parked); the "$T_W = 0$" cases refer to the estimation that has a strict separation of short-term parking, but with zero extra wait time; in this case, short term parking is only used by cars that are directly matched between the end and start of consecutive trips. In this case, "only LT" includes the count of only long-term parking demand, while "ST and LT" includes the sum of long-term and short-term parking demand. Figures were created with Gnuplot, version 5.2, available at `http://gnuplot.info`.

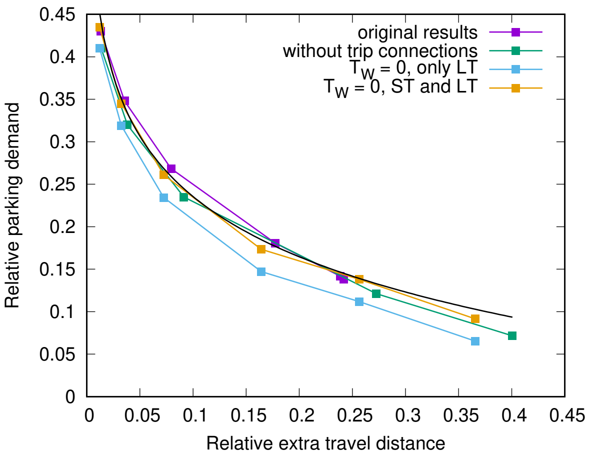

Figure S12: Relative parking demand as a function of extra VKT calculated with different approaches. We see that all approaches show very similar results that are consistent with the exponential fit in the main text (black line); the main difference is the case when we disregard short-term parking ("$T_W = 0$, only LT"), which is a clear underestimation of total parking requirements. Figure was created with Gnuplot, version 5.2, available at `http://gnuplot.info`.

The analysis above provides an approximate lower bound on parking provision, since the number of spaces for many uses is estimated based on *minimum* parking requirements; limits on maximum parking in Singapore were established since February 2019, and thus do not apply to the buildings considered in our study yet [12]. In accordance with the goverment's aim to be "car-lite", minimum parking requirements in Singapore are quite low, up to 5 to 10 times lower than those in use in the USA [14] for certain commercial uses. However, some developers may have voluntarily provided parking in excess of these minimums. Therefore, we adjust the distribution of parking spaces upwards based on trip data from SimMobility. We do this by performing a simplified estimate for the current trips made in private cars, where we require everyone to be able to park at the start and end location of their trips exactly (similarly to Algorithm 1 in our previous work [1], but with zero search radius). We note the trip start and end locations were aggregated to a set of 4,529 discrete locations, which can be interpreted as potential locations for car parks. To combine these results with the previous estimation, we map the building locations to all possible trip start locations in a 250 m radius around them, and distribute the estimated parking in a way that minimizes the discrepancy between the two results. After this, for each location, we take the larger value from the two estimates as our final estimate on parking there. The main idea behind this is to adjust estimates in locations where basing them only on minimum parking requirements gives unrealistically low values; this is the case especially for office buildings in the CBD area, which is the target of a significantly high number of commuting trips. After these adjustments, we arrive at a number of 1,369,576 parking places, or 2.02 per person in our simulation. We note that this is still a relatively low number, thus we can assume that this is a lower bound on the real number of parking in Singapore. We display the spatial distribution of parking spaces obtained with this method in Fig. S13.

5 Spatial distribution of parking demand

Our analysis allows us to investigate not only fleet size and aggregate parking needs, but also the spatial distribution of current parking supply and future demand if OVs are adopted. We display these distributions in Fig. S13 (estimation of current supply) and S14 (estimation parking dmenad of OVs). Furthermore, we display the difference between the two (i.e. the possible savings due to adoption of OVs) in Fig. S15. The main difference is that while the current distribution of parking spaces is quite spread out over the dense areas of Singapore, with significant parking provisions for residential areas, the parking demand of the OV fleet is highly concentrated in the CBD, especially for low values of $r_{\max}$ with a few smaller peaks in residential centers in Punggol, Woodlands, and Pasir Ris.

A main contributing factor to this spatial asymmetry is the high concentration of jobs in the CBD, thus the imbalance of commuting flows going there. Increasing $r_{\max}$ results in a significant decrease in the parking demand in the city center in our estimation; this is understandable, since a higher $r_{\max}$ allows OVs bringing workers to the centre to choose a parking location during the day in a much larger area. On the other hand, in residential areas, there is a potential oversupply of parking even in the current situation, with a total of 962 thousand parking spaces currently supplied for primarily residential use, or 1.42 residential parking spaces per private car user. We note that many of these parking spaces are actually usable by the general public,

use type	total demand	parking requirement	total parking
public housing (HDB)	1,188,649 units	N/A	640,188
private housing	321,974 units	1 / unit	321,974
office (zone 1)	4,362,915 m^2	1 / 450 m^2	9,695
office (zone 2)	1,590,091 m^2	1 / 250 m^2	6,360
office (zone 3)	1,280,454 m^2	1 / 200 m^2	6,402
retail (zone 1)	1,657,742 m^2	1 / 400 m^2	4,144
retail (zone 2)	1,812,934 m^2	1 / 200 m^2	9,065
retail (zone 3)	1,729,218 m^2	1 / 150 m^2	11,528
factory	34,909,726 m^2	1 / 450 m^2	77,577
other	23,648,697 m^2	1 / 300 m^2	78,829
URA parking lots	N/A	N/A	25,740
Total estimated			1,191,502
Total used in trips			1,064,952
Combined estimate			1,369,576

Table S4: Summary of estimates of different types of parking in Singapore. Parking is given as an aggregate number for housing managed by the HDB, while it is calculated based on minimum requirements for all other types. Office and retail uses have different parking requirements in three city zones. Zone 1 is defined as the central business district and its vicinity, zone 2 is defined as the area within 400 m distance from any rapid transit station, while zone 3 is the rest of the city. We used the "retail" category for any commercial establishment, while the minimum parking requirement for the "factory" and "other" categories were determined as the average of multiple relevant categories.

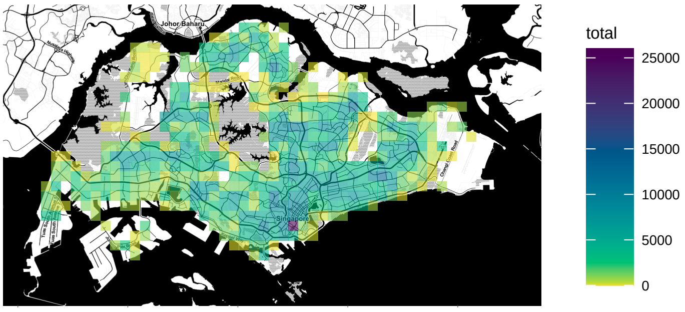

Figure S13: Spatial distribution of parking spaces as estimated from the SimMobility building and trip database. The total number of parking spaces is 1.37 million. Resolution of the grid displayed is 1 km × 1 km similarly to Fig. S14, i.e. the numbers given are parking per square kilometer. Note that the color scale is the same as in Fig. S14. Map data by OpenStreetMap contributors, available under the ODbL License [15]. Map tiles by Stamen design, available under CC BY 3.0 [16]. Figure generated by using the `ggmap` [17], and `colorspace` [18] R packages.

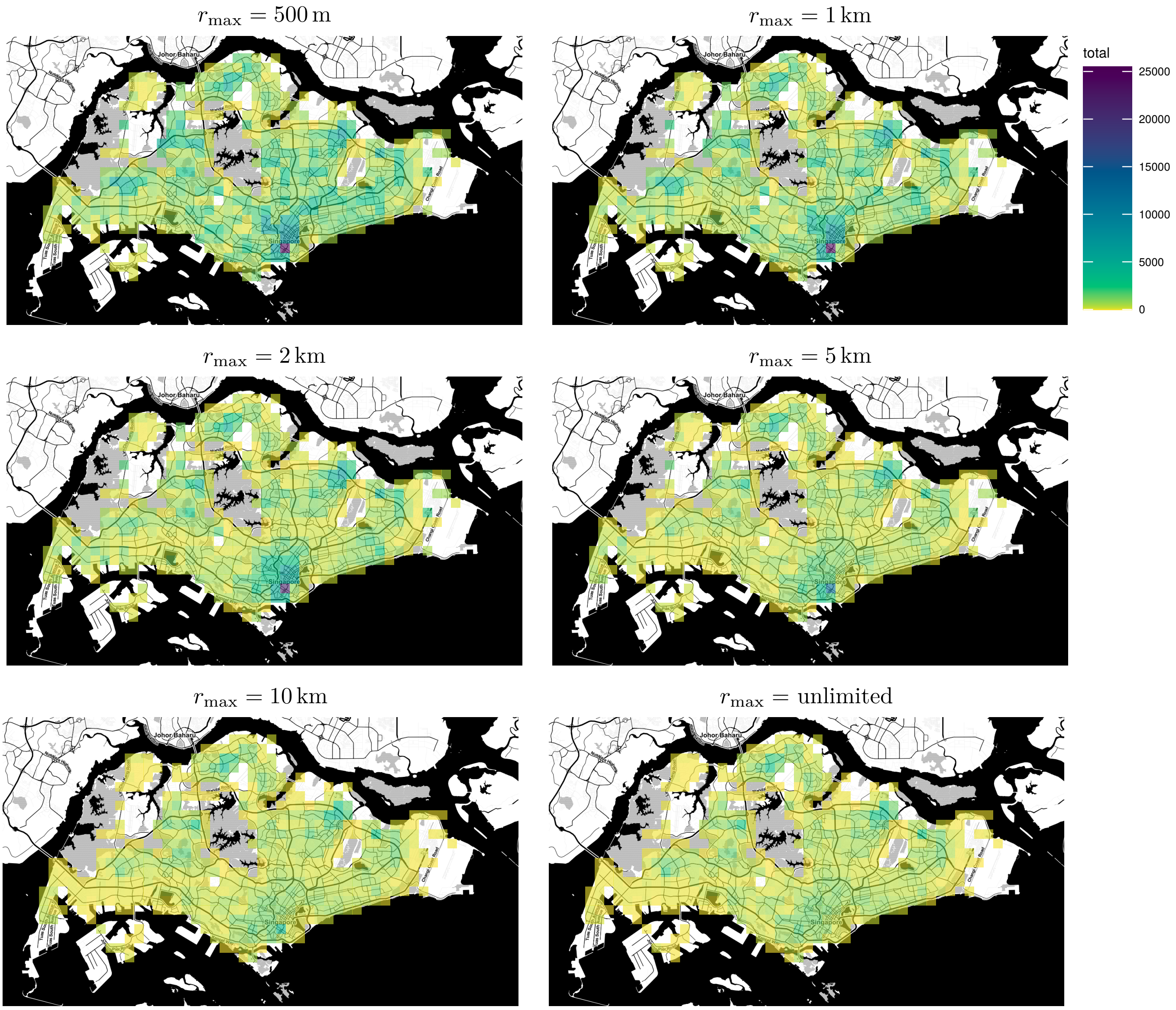

Figure S14: Spatial distribution of parking demand found as a result of the batched bipartite matching estimation, i.e. Algorithm 2. Resolution of the grid displayed is 1 km × 1 km, i.e. the number of parking spaces are given as per square kilometer. Results are displayed for $r_{\max}$ values between 500 m and unlimited. Note that the color scale on all panels is the same, along with the colors used in Fig. S13. All grid cells have less than 10,000 parking spaces with the exception of one outlier in the central business district that has 24,723, 24,313, 21,326 and 11,577 parking spaces for $r_{\max} = 500\,\mathrm{m}$, 1 km, 2 km and 5 km respectively. Map data by OpenStreetMap contributors, available under the ODbL License [15]. Map tiles by Stamen design, available under CC BY 3.0 [16]. Figure generated by using the `ggmap` [17], and `colorspace` [18] R packages.

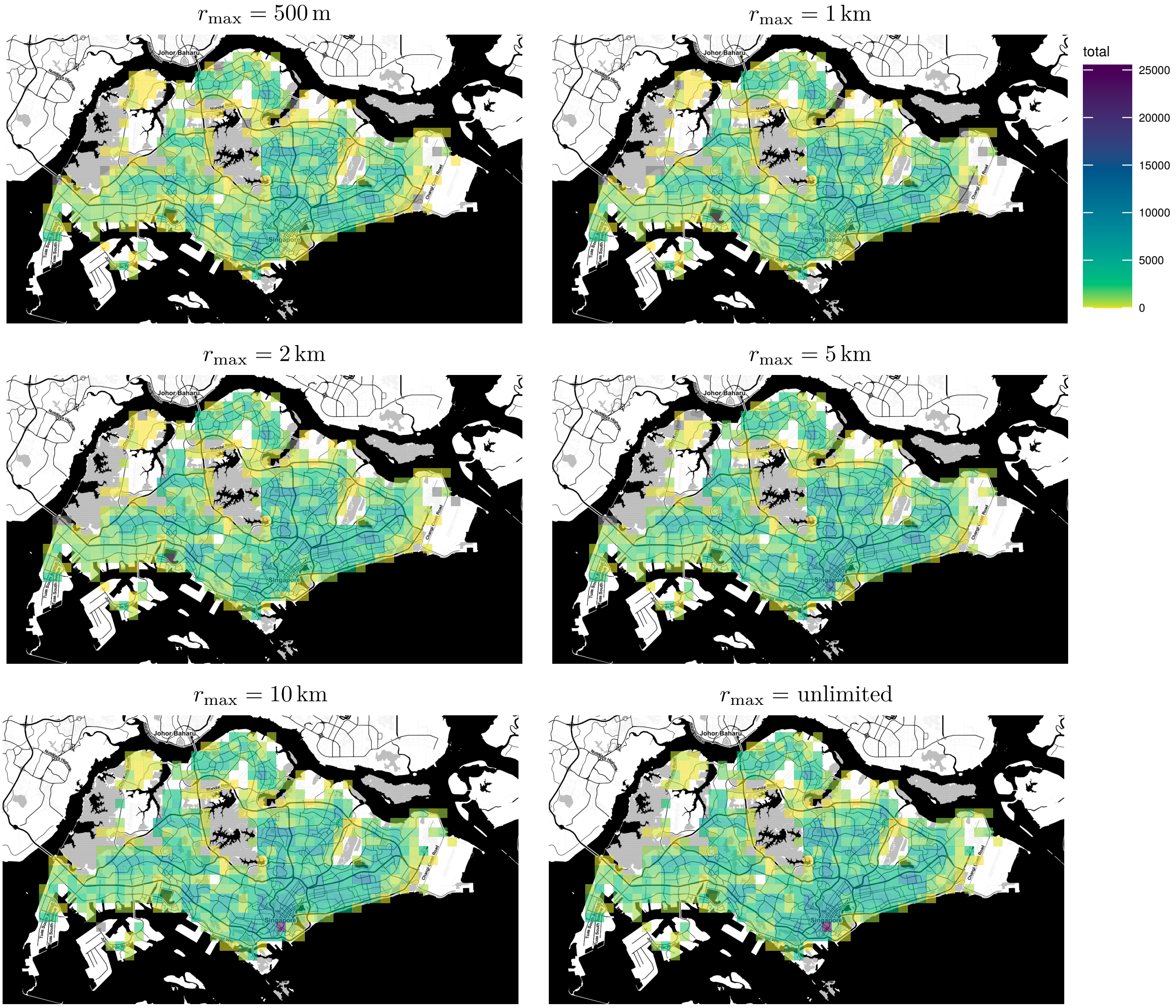

Figure S15: Spatial distribution of savings in parking demand, i.e. the difference between the spatial distributions displayed in Figs. S14 and S13. Map data by OpenStreetMap contributors, available under the ODbL License [15]. Map tiles by Stamen design, available under CC BY 3.0 [16]. Figure generated by using the `ggmap` [17], and `colorspace` [18] R packages.

i.e. parking managed by the Housing Development Board (HDB) of Singapore.

Looking at the differences in Fig. S15, we see that most areas can be expected to experience a significant decrease of parking demand; this is most prominent in dense residential areas such as Bedok, Toa Payoh, Ang Mo Kio or Clementi, where the decrease in demand is already significant for low $r_{\max}$ values. A significant amount of parking in these settings is provided by the HDB as surface parking lots and separate parking garages, thus repurposing the land area taken up by these could be more easily achieved than underground parking facilities found with many commercial and private residential buildings.

This is even more evident when looking at actual parking use during the night and day (see Fig. S16 and the Supplementary Videos provided displaying the utilization of parking during the day), showing a significant difference in the use of parking , with night usage spread in residential neighborhoods across Singapore and day usage concentrated in the CBD and a few areas with a high number of workplaces such as the Nanyang Technological University Campus, Tuas or Changi.

References

[1] D. Kondor, H. Zhang, R. Tachet, P. Santi, and C. Ratti, "Estimating savings in parking demand using shared vehicles for home-work commuting," *IEEE Transactions on Intelligent Transportation Systems*, 2018.

[2] G. S. Bauer, J. B. Greenblatt, and B. F. Gerke, "Cost, Energy, and Environmental Impact of Automated Electric Taxi Fleets in Manhattan," *Environmental Science and Technology*, vol. 52, no. 8, pp. 4920–4928, 2018.

[3] M. M. Vazifeh, P. Santi, G. Resta, S. H. Strogatz, and C. Ratti, "Addressing the minimum fleet problem in on-demand urban mobility," *Nature*, vol. 557, pp. 534–538, 2018.

[4] J. E. Hopcroft and R. M. Karp, "An $n^{\frac{5}{2}}$ algorithm for maximum matching in bipartite graphs," *SIAM Journal on Computing*, vol. 2, no. 4, pp. 225–231, 1973. [Online]. Available: https://doi.org/10.1137/0202019

[5] F. T. Boesch and J. F. Gimpel, "Covering Points of a Digraph with Point-Disjoint Paths and Its Application to Code Optimization," *Journal of the ACM*, vol. 24, no. 2, pp. 192–198, 1977. [Online]. Available: http://portal.acm.org/citation.cfm?doid=322003.322005

[6] P. Santi, G. Resta, M. Szell, S. Sobolevsky, S. H. Strogatz, and C. Ratti, "Quantifying the benefits of vehicle pooling with shareability networks," *PNAS*, vol. 111, no. 37, pp. 13 290–13 294, 2014. [Online]. Available: https://doi.org/10.1073/pnas.1403657111

[7] R. Tachet, O. Sagarra, P. Santi, G. Resta, M. Szell, S. H. Strogatz, and C. Ratti, "Scaling law of urban ride sharing," *Scientific Reports*, vol. 7, p. 42868, 2017. [Online]. Available: http://dx.doi.org/10.1038/srep42868

[8] S. L. Authority, "Onemap.sg," https://www.onemap.sg/, 2019, postal code data accessed on 2019-11-29 from OneMap.sg which is made available under the terms of the Singapore Open Data Licence version 1.0 (https://www.onemap.sg/legal/opendatalicence.html).

[9] D. Sim, "`singapore-postal-codes` – Data dump of Singapore postal codes from scraping Onemap.sg," https://github.com/xkjyeah/singapore-postal-codes, 2019, accessed: 2019-11-29.

[10] "Properties and Facilities Under HDB Management – Car Parks Under HDB Management," https://data.gov.sg/dataset/hdb-properties-facilities-under-management?resource_id=78c18b2b-b4cb-47d7-a953-612b3a2800ce, 2019, accessed: 2019-08-07.

[11] "Code of Practice: Vehicle Parking Provision in Development Proposals," https://www.lta.gov.sg/content/dam/ltaweb/corp/Industry/files/VPCOP2011.pdf, 2011, accessed: 2019-08-07.

[12] LTA, "New Parking Standards for Private Developments from February 2019," https://www.lta.gov.sg/content/ltagov/en/newsroom/2018/11/2/new-parking-standards-for-private-developments-from-february-2019.html, 2018, accessed: 2020-02-10.

[13] "URA Parking Lots," https://data.gov.sg/dataset/ura-parking-lot?resource_id=6baa1ff2-a647-41a0-bb7e-17218964e91e, 2019, accessed: 2019-08-07.

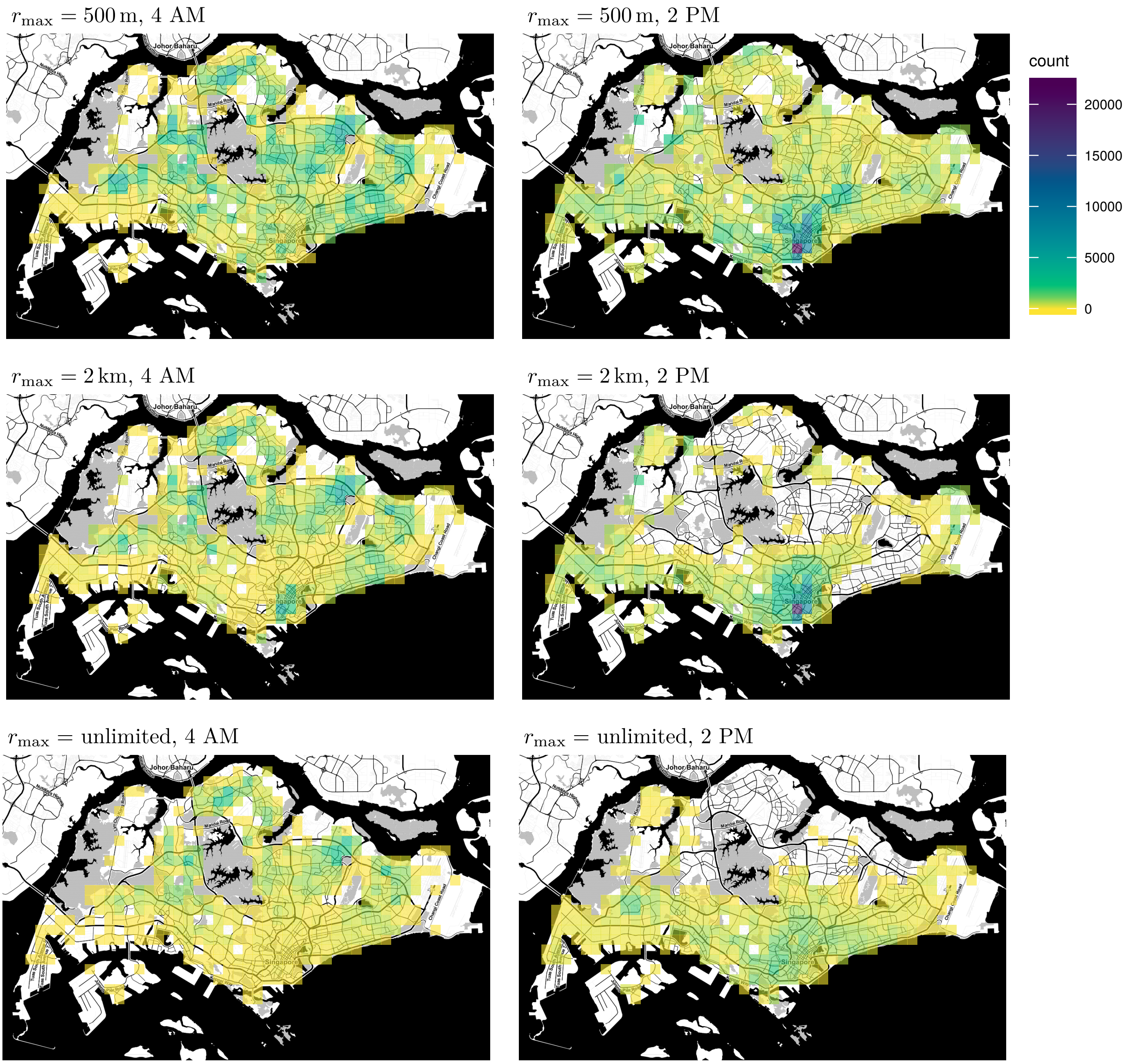

Figure S16: Spatial distribution of actual parking usage at night (4 AM, in the left column) and midday (2 PM, in the right column) for $r_{\max} = 500\,\text{m}$, $2\,\text{km}$ and unlimited in the top, middle and bottom rows respectively. It is clear that parking usage shows a large difference during night and day, in accordance with the imbalance of commuting flows. In theory, cars could go back to park in residential neighborhoods during the day, at the cost of even more empty miles traveled (see Section 3.3 and Fig. S11 for a discussion of this in more detail). Map data by OpenStreetMap contributors, available under the ODbL License [15]. Map tiles by Stamen design, available under CC BY 3.0 [16]. Figure generated by using the `ggmap` [17], and `colorspace` [18] R packages.

[14] M. Chester, A. Fraser, J. Matute, C. Flower, and R. Pendyala, "Parking Infrastructure: A Constraint on or Opportunity for Urban Redevelopment? A Study of Los Angeles County Parking Supply and Growth," *Journal of the American Planning Association*, vol. 81, no. 4, pp. 268–286, 2015. [Online]. Available: http://www.tandfonline.com/doi/full/10.1080/01944363.2015.1092879

[15] "Openstreetmap data available under the ODbL License," https://www.openstreetmap.org/copyright, accessed: 2020-08-17.

[16] "Map tiles by Stamen Design, available under CC BY 3.0." https://maps.stamen.com, accessed: 2020-08-17.

[17] D. Kahle and H. Wickham, "ggmap: Spatial visualization with ggplot2," *The R Journal*, vol. 5, no. 1, pp. 144–161, 2013. [Online]. Available: https://journal.r-project.org/archive/2013-1/kahle-wickham.pdf

[18] A. Zeileis, K. Hornik, and P. Murrell, "Escaping RGBland: Selecting colors for statistical graphics," *Computational Statistics & Data Analysis*, vol. 53, no. 9, pp. 3259–3270, 2009. [Online]. Available: https://doi.org/10.1016/j.csda.2008.11.033
